# A polygenic risk score derived from common variants of monogenic diabetes genes is associated with young-onset type 2 diabetes and cardiovascular–kidney complications

**DOI:** 10.1007/s00125-024-06320-3

**Published:** 2024-11-23

**Authors:** Chun-Kwan O, Baoqi Fan, Sandra T. F. Tsoi, Claudia H. T. Tam, Raymond Wan, Eric S. H. Lau, Mai Shi, Cadmon K. P. Lim, Gechang Yu, Jane P. Y. Ho, Elaine Y. K. Chow, Alice P. S. Kong, Risa Ozaki, Wing Yee So, Ronald C. W. Ma, Andrea O. Y. Luk, Juliana C. N. Chan

**Affiliations:** 1 Department of Medicine and Therapeutics, Faculty of Medicine, The Chinese University of Hong Kong, Prince of Wales Hospital, Hong Kong, China; 2 Li Ka Shing Institute of Health Sciences, The Chinese University of Hong Kong, Prince of Wales Hospital, Hong Kong, China; 3 Hong Kong Institute of Diabetes and Obesity, The Chinese University of Hong Kong, Prince of Wales Hospital, Hong Kong, China

**Keywords:** Complications, Genetics, MODY, Polygenic risk scores, Whole-exome sequencing, Young-onset diabetes

## Abstract

**Aims/hypothesis:**

Monogenic diabetes is caused by rare mutations in genes usually implicated in beta cell biology. Common variants of monogenic diabetes genes (MDG) may jointly influence the risk of young-onset type 2 diabetes (YOD, diagnosed before the age of 40 years) and cardiovascular and kidney events.

**Methods:**

Using whole-exome sequencing data, we constructed a weighted polygenic risk score (wPRS) consisting of 135 common variants (minor allele frequency >0.01) of 34 MDG based on *r*^2^>0.2 for linkage disequilibrium in a discovery case–control cohort of 453 adults with YOD (median [IQR] age 39.7 [34.9–46.9] years) and 405 without YOD (median [IQR] age 56.7 [50.3–61.0] years), followed by validation in an independent cross-sectional cohort with array-based genotyping for YOD and a prospective cohort of individuals with type 2 diabetes for cardiovascular and kidney events.

**Results:**

In the discovery cohort, the OR of the 135 common variants for YOD ranged from 1.00 to 2.61. In the validation cohort (920 YOD and 4910 non-YOD), top-10%-wPRS was associated with an OR of 1.42 (95% CI 1.03, 1.95, *p*=0.033) for YOD compared with bottom-10%-wPRS. In 2313 individuals with type 2 diabetes (median [IQR]: age 53.4 [45.4–61.7] years; disease duration 4.0 [1.0–9.0] years) observed for a median (IQR) of 17.5 (14.4–21.8) years, standardised wPRS was associated with increased HR for incident cardiovascular events (1.16 [95% CI 1.06, 1.27], *p*=0.001), kidney events (1.09 [95% CI 1.02, 1.16], *p*=0.013) and cardiovascular–kidney events (1.10 [95% CI 1.03, 1.16], *p*=0.003). Using the ‘bottom-20%-wPRS plus baseline disease duration <5 years’ group as referent, the ‘top-20%-wPRS plus baseline disease duration 5 to <10 years’ group had unadjusted and adjusted HR of 1.60 (95% CI 1.17, 2.19, *p*=0.003) and 1.62 (95% CI 1.16, 2.26, *p*=0.005), respectively, for cardiovascular–kidney events compared with 1.38 (95% CI 0.97, 1.98, *p*=0.075) and 1.06 (95% CI 0.72, 1.57, *p*=0.752) in the ‘bottom-20%-wPRS plus baseline disease duration ≥10 years’ group.

**Conclusions/interpretation:**

Common variants of MDG increased risk for YOD and cardiovascular–kidney events.

**Graphical Abstract:**

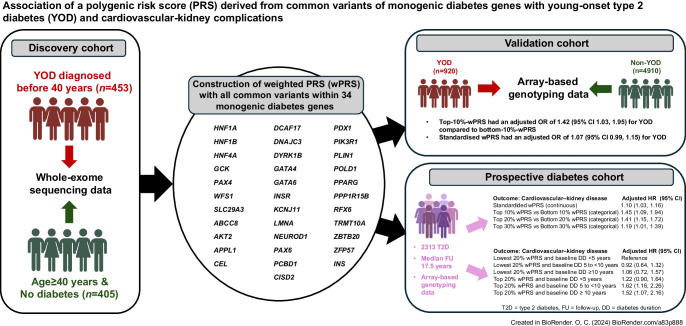

**Supplementary Information:**

The online version contains peer-reviewed but unedited supplementary material available at 10.1007/s00125-024-06320-3.


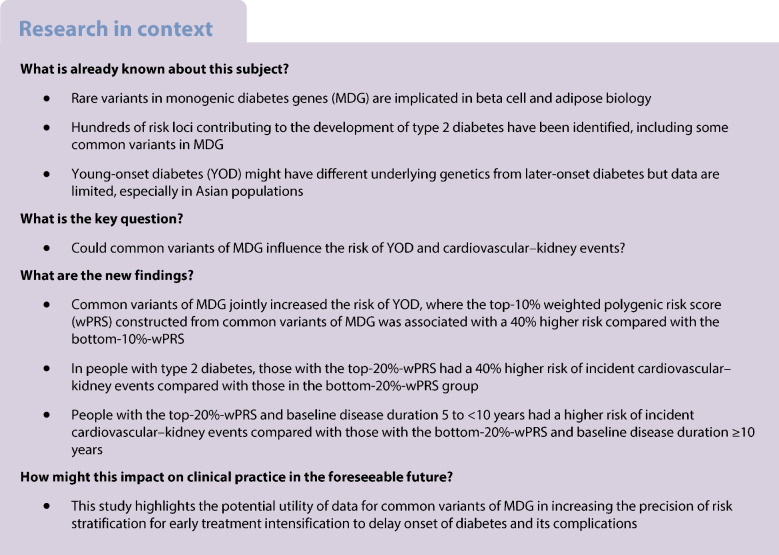


## Introduction

Monogenic diabetes, including MODY, refers to a group of diabetes of Mendelian inheritance due to rare mutations of specific genes usually implicated in developmental and beta cell biology, glucose sensing and insulin translation or processing [1]. Some mutations cause severe insulin resistance due to dysregulation of insulin signalling or fat metabolism, while some are associated with syndromic features [2]. The physiological roles of monogenic diabetes genes (MDG) are supported by experimental animal models and/or co-segregation among family members [1–3]. Given their functional significance, we argue that their common variants, albeit with smaller effect size, might contribute to young onset of type 2 diabetes. Apart from their effects on energy metabolism, the high glycaemic burden resulting from potentially poor glycaemic trajectory and long duration of exposure to abnormal milieu due to younger age at diabetes onset will increase the risk of cardiovascular–kidney events [4].

Large-scale genome-wide association studies (GWAS) across ancestries have reported associations of common variants of MDG with type 2 diabetes or related traits [5, 6]. Given potential differences in the aetiologies of young-onset diabetes (YOD, diagnosed before the age of 40 years) and later-onset diabetes (LOD), a few studies revealed specific genetic associations stratified by age of diagnosis [7, 8]. In the first GWAS targeting youth-onset diabetes in a multi-ethnic cohort (non-Hispanic White, African American and Hispanic) with an age of diagnosis <20 years and a mean age of 15 years, researchers identified seven genome-wide significant loci, including one novel signal in *PHF2* (encoding for PHD finger protein 2) not reported to be associated with type 2 diabetes in adults [9]. However, similar genetic studies for YOD diagnosed before the age of 40 years are lacking, especially in Asians.

We hypothesised that common variants of MDG jointly increase the risk of YOD and incident cardiovascular–kidney complications, and tested this hypothesis by constructing a weighted polygenic risk score (wPRS) with common variants of 34 MDG based on a discovery cohort (YOD vs non-YOD) with whole-exome sequencing (WES), followed by validation in independent cohorts with genotyping.

## Methods

### Study design and participants

**Fig. 1:**
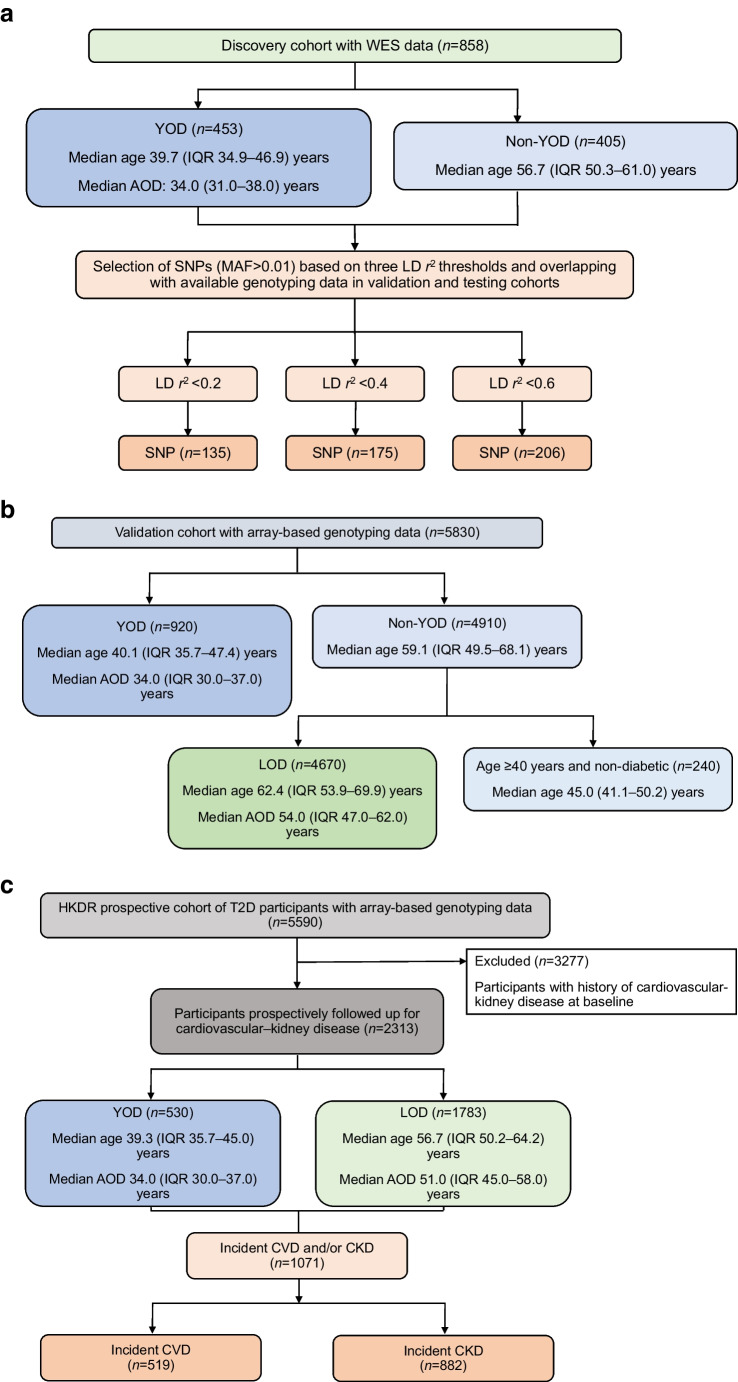
Discovery cohort, validation cohort and prospective HKDR cohort. (**a**) Discovery cohort and selection of SNPs within MDG for construction of polygenic risk scores for YOD. All non-YOD participants were aged ≥40 years and without diabetes. (**b**) Validation cohort for assessing performance of wPRS for YOD. (**c**) Prospective HKDR cohort for assessing association between wPRS and incident cardiovascular–kidney complications in type 2 diabetes. AOD, age of diagnosis; MAF, minor allele frequency; T2D, type 2 diabetes

Participants came from three established cohorts (Fig. 1 and electronic supplementary material [ESM] Fig. 1): the Hong Kong Family Diabetes Study (HKFDS); Better Health for Better Hong Kong (BHBHK); and the Hong Kong Diabetes Register (HKDR).

The HKFDS cohort was established in 1998–2003 by the Chinese University of Hong Kong (CUHK) Diabetes Care and Research Team. There were 192 index cases with diabetes (149 with YOD), and their family members, giving a total of 1076 participants, recruited for studying genetic and environmental causes of diabetes in the Chinese population [10, 11]. The index cases were identified in the diabetes complication assessment programme at the Prince of Wales Hospital (PWH), followed by invitation of their relatives to participate in the study.

The BHBHK cohort was established in 2001–2003 as part of a community-based health promotion campaign to screen for cardiovascular risk factors including obesity and diabetes in the workforce [12]. The HKFDS cohort and a random BHBHK sub-cohort (*n*=863) underwent structured assessment, including personal and family history, anthropometric measurements and collection of blood and urine samples for metabolic profiling [11, 13, 14]. They underwent a 2 h 75 g OGTT with measurements of plasma insulin, C-peptide and glucose, accompanied by a DNA/serum biobank. In 2012–2014, the diabetes status of both cohorts was ascertained using medical records, OGTT and HbA_1c_ [11].

The HKDR was established in 1995 by the CUHK-PWH Team as a research-driven quality improvement programme in a hospital-based setting. Patients with diabetes could be referred from all PWH medical clinics to the PWH Diabetes Centre where collection of clinical information, screening for diabetes-related complications, and data management and reporting were conducted, guided by a pre-defined protocol [15]. The participants were prospectively observed with ascertainment of clinical outcomes retrieved from the territory-wide electronic medical record system of the Hong Kong Hospital Authority. Details of the rationale, setting, team structure, procedures, database management and data-driven care were reported [16]. These studies were approved by the CUHK Clinical Research Ethics Committee.

### Discovery cohort, validation cohort and HKDR prospective cohort

The discovery cohort consisted of 453 individuals with YOD from the HKDR and 405 individuals without YOD from the BHBHK with WES as part of the Global Type 2 Diabetes Consortium [17]. ESM Tables 1–2 show the variants within the 34 MDG. ESM Table 3 summarises the methodology for analysing WES and array-based genotyping data used to construct a wPRS.

We validated the performance of the derived wPRS in predicting YOD in a separate cohort of 920 individuals with YOD from the HKDR and 4910 individuals without YOD from the HKDR and the BHBHK (excluding those involved in discovery cohort) with available array-based genotyping data (validation cohort). The non-YOD group included 4670 individuals with LOD from the HKDR and 240 without diabetes from the BHBHK.

We tested the association between wPRS and incident cardiovascular–kidney events in the prospective HKDR cohort of 2313 Chinese individuals with type 2 diabetes stratified by wPRS deciles and disease duration (excluding those with CVD, kidney disease and albuminuria at baseline, and those involved in discovery cohort). In a secondary analysis, we tested the associations of the wPRS with beta cell function indices and incident diabetes in 363 individuals without diabetes at baseline from the BHBHK and the HKFDS (excluding those involved in the discovery cohort, randomly picking one individual from each family for HKFDS).

### Definitions and outcomes

In all cohorts, sex referred to the biological sex of the individuals, and the information was defined by the sex entity recorded in official government documents such as the Hong Kong Identity Card. In the discovery cohort, YOD was defined as diabetes diagnosed before the age of 40 years and non-YOD was defined as no diabetes at the age of ≥40 years. In the validation cohort, we expanded the definition of the non-YOD group to include LOD diagnosed at age ≥40 years for a larger sample size.

In the prospective HKDR cohort, incident cardiovascular–kidney complications were defined by hospital discharge principal diagnoses and procedures coded by ICD-9 (http://www.icd9data.com/2007/Volume1/default.htm) and laboratory variables: (1) CHD; (2) stroke; (3) peripheral vascular disease (PVD); (4) congestive heart failure (CHF); (5) CVD; (6) chronic kidney disease (CKD); (7) end-stage kidney disease (ESKD); and (8) composite cardiovascular–kidney disease (ESM Table 4).

In the secondary analysis, we examined the associations of the wPRS with incident diabetes and beta cell function indices in individuals without diabetes at baseline. We calculated HOMA2-%B and HOMA2-IR using the HOMA2 calculator v2.2.3 (https://www.dtu.ox.ac.uk/homacalculator/). Insulinogenic index and disposition index as indices of beta cell function and insulin resistance were calculated as follows:

$$\text{Insulinogenic index }= \frac{\mathrm{Ins}30-\mathrm{Ins}0}{\mathrm{Gluc}30-\mathrm{Gluc}0}$$

$$\text{Disposition index }= \frac{\text{Insulinogenic index}}{6 \times \mathrm{HOMA}-\mathrm{IR}}$$

where Ins0, Ins30, Gluc0 and Gluc30 are plasma insulin at 0 min, insulin at 30 min, glucose at 0 min and glucose at 30 min during OGTT, respectively (units for plasma insulin and glucose are pmol/l and mmol/l, respectively).

### Construction of wPRS

The wPRS was constructed as follows:

$$\mathrm{wPRS }= {\upbeta }_{1\times 1} + {\upbeta }_{2\times 2} + _{\dots} + {\upbeta }_{\mathrm{k}}{\mathrm{x}}_{\mathrm{k}} + _{\dots} + {\upbeta }_{\mathrm{n}}{\mathrm{x}}_{\mathrm{n}}$$

where β_k_ is the per-allele effect size for YOD associated with a single-nucleotide variant k of the 34 MODY genes, x_k_ is the number of effect alleles of the single-nucleotide variant k, and n is the total number of single-nucleotide variants involved in the construction of the polygenic risk score.

We employed a ‘pruning-and-thresholding’ approach to construct and choose a wPRS with optimal performance. Due to use of different arrays, we selected SNPs available in all cohorts and included 135, 175 and 206 SNPs located within ±1000 base-pairs of gene regions of the 34 MDG by varying *r*^2^ thresholds of linkage disequilibrium (LD) at 0.2, 0.4 and 0.6, respectively.

### Statistical analysis

All data were expressed as mean ± SD or median (IQR). Between-group comparisons were made by parametric and non-parametric tests as appropriate. The wPRS was transformed into a standardised wPRS (swPRS, see equation below) or divided into categories in the association analyses.

$$\mathrm{swPRS}= \frac{\text{wPRS of a specific individual}-\text{mean wPRS}}{\text{SD of wPRS}}$$

Binary logistic regression was used to examine the association of the wPRS with YOD expressed as OR with 95% CI in the validation cohort. Kaplan–Meier estimation accompanied by curves of one-minus-survival functions was used to describe the cumulative incidence of events with logranked test for examining differences among groups in the prospective HKDR cohort. Cox proportional hazard regression was used to examine the association of the wPRS with incident cardiovascular–kidney events expressed as HR with 95% CI accompanied by curves of one-minus-survival functions with covariates controlled at mean for continuous variables and at reference category for nominal variables. In the secondary analysis, we randomly picked one individual from each family and used multivariate linear and binary logistic regression to examine the associations of the wPRS with beta cell function indexes (HOMA-2%B, insulinogenic index, disposition index), insulin resistance (HOMA2-IR) and incident diabetes in the BHBHK-HKFDS. Missing data were handled by pairwise deletion, and the total number of individuals involved in each model of regression analysis were stated.

## Results

### Construction and validation of the wPRS for YOD

Figure 1 and Table 1 summarise the profiles of the discovery and validation cohorts, and the prospective HKDR cohort at baseline. We analysed the WES data of 453 individuals with YOD from the HKDR (median age 39.7 [IQR 34.9–46.9] years; median age of diagnosis 34.0 [IQR 31.0–38.0] years) and 405 individuals without YOD (non-YOD) from the BHBHK (median age 56.7 [IQR 50.3–61.0] years) and estimated the effect size of each SNP of the 34 MDG for YOD (discovery cohort). Using overlapping genotyping data from the BHBHK, HKFDS and HKDR, we selected 135, 175 and 206 SNPs to construct three wPRS using LD statistics *r*^2^ thresholds of 0.2, 0.4 and 0.6, respectively.

**Table 1:** Baseline characteristics of the discovery, validation and prospective HKDR cohorts

Characteristic	Discovery cohort for wPRS construction^a^						Validation cohort for YOD^b^				Prospective HKDR cohort for testing relationship of wPRS with incident cardiovascular–kidney events^b^	
	YOD (*n*=453)		Non-YOD (*n*=405)		YOD (*n*=920)		Non-YOD (*n*=4910)				YOD and LOD (*n*=2313)	
	*n*	Observations	*n*	Observations	*n*	Observations	LOD (*n*=4670)		No diabetes (*n*=240)		*n*	Observations
							*n*	Observations	*n*	Observations		
YOD	453	453 (100)	405	0 (0)	920	920 (100)	4670	0 (0)	240	0 (0)	2313	530 (22.9)
LOD	453	0 (0)	405	0 (0)	920	0 (0)	4670	4670 (100)	240	0 (0)	2313	1783 (77.1)
Without diabetes	453	0 (0)	405	405 (100)	920	0 (0)	4670	0 (0)	240	240 (100)	2313	0 (0)
Age, years	453	39.7 (34.9–46.9)	405	47.6 (42.3–54.0)^c^	920	40.1 (35.7–47.4)	4670	62.4 (53.9–69.9)	240	45.0 (41.1–50.2)^d^	2313	53.4 (45.4–61.7)
Age of diagnosis, years	451	34.0 (31.0–38.0)			920	34.0 (30.0–37.0)	4670	54.0 (47.0–62.0)			2313	47.0 (40.0–55.0)
Men	453	224 (49.4)	405	213 (52.6)	920	331 (36.0)	4670	2180 (46.7)	240	101 (42.1)	2313	1050 (45.4)
Tobacco use	452				917		4660		190		2306	
Current smoker		78 (17.3)				134 (14.6)		574 (12.3)		15 (7.9)		329 (14.3)
Ex-smoker		40 (8.8)				80 (8.7)		890 (19.1)		8 (4.2)		289 (12.5)
Non-smoker		334 (73.9)				703 (76.7)		3196 (68.6)		167 (87.9)		1688 (73.2)
Alcohol use	452				914		4648		189		2295	
Current drinker		44 (9.7)				88 (9.6)		404 (8.7)		26 (13.8)		262 (11.4)
Ex-drinker		38 (8.4)				65 (7.1)		631 (13.6)		1 (0.5)		214 (9.3)
Non-drinker		370 (81.9)				761 (83.3)		3613 (77.7)		162 (85.7)		1819 (79.3)
BMI, kg/m^2^	453	25.2 (22.5–28.6)	405	24.0 (22.1–26.6)	919	25.1 (22.4–28.1)	4640	24.8 (22.5–27.2)	240	23.0 (21.2–25.6)	2308	24.5 (22.2–27.0)
Systolic BP, mmHg	453	124 (114–137)	405	119 (109–130)	920	123 (113–136)	4667	136 (124–151)	240	118 (106–128)	2313	129 (117–140)
Diastolic BP, mmHg	453	76 (69–82)	405	75 (70–82)	920	74 (68–81)	4665	76 (68–83)	240	74 (68–80)	2312	75 (68–82)
DD, years	451	6.0 (1.0–12.0)			920	7.0 (1.0–13.0)	4670	6.0 (2.0–11.0)			2313	4.0 (1.0–9.0)
<5 years		205 (45.5)				367 (39.9)		2041 (43.7)				1213 (52.4)
≥ 5 years and <10 years		89 (19.7)				188 (20.4)		1158 (24.8)				565 (24.4)
≥ 10 years		157 (34.8)				365 (39.7)		1471 (31.5)				535 (23.1)
HbA_1c_, mmol/mol	451	57 (45–72)			920	58 (46–74)	4670	56 (46–69)			2313	54 (44–66)
HbA_1c_, %	451	7.4 (6.3–8.7)			920	7.5 (6.4–8.9)	4670	7.3 (6.4–8.5)			2313	7.1 (6.2–8.2)
Fasting plasma glucose, mmol/l	453	7.8 (6.5–10.4)	404	4.9 (4.6–5.2)	920	8.0 (6.4–10.5)	4663	7.8 (6.4–9.9)	239	4.9 (4.6–5.2)	2312	7.7 (6.4–9.5)
LDL-cholesterol, mmol/l	453	3.1 (2.5–3.7)	285	3.1 (2.6–3.8)	843	3.0 (2.3–3.6)	4460	3.0 (2.5–3.7)	239	3.0 (2.5–3.7)	2223	3.1 (2.5–3.6)
HDL-cholesterol, mmol/l	453	1.2 (1.0–1.4)	405	1.4 (1.2–1.7)	905	1.3 (1.1–1.5)	4622	1.3 (1.1–1.5)	240	1.5 (1.3–1.8)	2286	1.3 (1.1–1.6)
Triacylglycerol, mmol/l	453	1.3 (0.9–1.9)	405	1.2 (0.8–2.0)	913	1.2 (0.8–1.9)	4648	1.4 (1.0–2.1)	240	0.9 (0.7–1.4)	2300	1.2 (0.9–1.9)
eGFR, ml/min per 1.73m^2^	452	105 (92–114)	403	93 (81–103)	920	104 (88–114)	4670	78 (59–94)	240	95 (84–107)	2313	92 (80–103)

Summary statistics are expressed as median (IQR) or *n* (%)

^a^WES data

^b^Array-based genotyping data

^c^The median age of the non-YOD group in the discovery cohort here is based on the age of the participants in the BHBHK cohort when they were first recruited at baseline (2001–2003). Some individuals included in the non-YOD group had their ‘no diabetes’ status re-ascertained at 12 year follow-up. The median age of the non-YOD group based on the known oldest age of ascertained ‘no diabetes’ status of the participants was 56.7 (IQR 50.3–61.0) years

^d^The median age of the ‘no diabetes’ subgroup of the non-YOD group in the validation cohort here is based on the age of the participants in the BHBHK cohort when they were first recruited at baseline (2001–2003). Some individuals included in the non-YOD group had their ‘no diabetes’ status re-ascertained at 12 year follow-up. The median age of the ‘no diabetes’ subgroup based on the known oldest age of ascertained ‘no diabetes’ status of the participants was 54.7 (IQR 49.0–60.0) years

In the validation cohort, all swPRS were positively associated with increased odds for YOD. The wPRS constructed using SNPs with *r*^2^<0.2 performed the best, with the swPRS having an unadjusted OR of 1.073 (95% CI 1.00, 1.15, *p*=0.051) and a sex- and BMI-adjusted OR of 1.07 (95% CI 0.99, 1.15, *p*=0.074) for YOD (Table 2 and ESM Table 5a). The OR of 1.08 (95% CI 0.94, 1.25, *p*=0.280) remained similar in a sensitivity analysis with strict inclusion of only those aged ≥40 years with no diabetes as the non-YOD group (ESM Table 5b). Using these SNPs (*r*^2^<0.2), the top-10%-wPRS group had 42% higher risk of YOD than the bottom-10%-wPRS group (OR 1.42 [95% CI 1.03, 1.95], *p*=0.033) while the OR and significance (1.11 [95% CI 0.89, 1.39], *p*=0.346) were attenuated when comparing top-20% with bottom-20%-wPRS. The wPRS based on LD *r*^2^ threshold of 0.2 was therefore used in the subsequent analysis, and the OR of the 135 SNPs for YOD ranged from 1.00 to 2.61 in the discovery cohort (ESM Table 2b).

**Table 2:** Associations of wPRS, based on LD *r*^2^ threshold of 0.2 during selection of SNPs, with YOD in validation cohort of 920 individuals with YOD and 4910 individuals without YOD

Model	Continuous swPRS	Top 10% vs bottom 10% wPRS	Top 15% vs bottom 15% wPRS	Top 20% vs bottom 20% wPRS
Unadjusted				
*N*	5830	1166	1750	2332
OR (95% CI)	1.07 (1.00, 1.15)	1.43 (1.04, 1.97)	1.31 (1.02, 1.70)	1.12 (0.90, 1.40)
*p* value	0.051	0.026*	0.037*	0.308
Adjusted for PC1 and PC2				
*N*	5830	1166	1750	2332
OR (95% CI)	1.07 (0.99, 1.14)	1.42 (1.04, 1.96)	1.30 (1.01, 1.69)	1.12 (0.89, 1.40)
*p* value	0.077	0.029*	0.044*	0.330
Add-on adjustment for sex				
*N*	5830	1166	1750	2332
OR (95% CI)	1.07 (0.99, 1.14)	1.41 (1.03, 1.94)	1.31 (1.01, 1.69)	1.11 (0.89, 1.39)
*p* value	0.082	0.034*	0.044*	0.365
Add-on adjustment for BMI				
*N*	5799	1157	1738	2317
OR (95% CI)	1.07 (0.99, 1.15)	1.42 (1.03, 1.95)	1.31 (1.01, 1.70)	1.11 (0.89, 1.39)
*p* value	0.074	0.033*	0.041*	0.346

*p<0.05

PC1, first principal component; PC2, second principal component

### Association of the wPRS with incident cardiovascular–kidney events in individuals with type 2 diabetes

In the HKDR, 2313 individuals with no history of cardiovascular–kidney events and albuminuria at baseline (enrolled in 1994–2007) were identified. After a median follow-up of 17.5 (IQR 14.4–21.8) years, there was an accrual of 519 cardiovascular and 882 kidney events. Per-SD increase in wPRS was associated with an HR of 1.10 (95% CI 1.03, 1.16, *p*=0.003) for cardiovascular–kidney events (Table 3). The top-20%-wPRS group had 41% higher risk than the bottom-20%-wPRS group (HR 1.41 [95% CI 1.15, 1.72], *p*<0.001) after adjusting for baseline demographics, metabolic control (BMI, HbA_1c_, systolic BP, triacylglycerol, LDL-cholesterol and HDL-cholesterol), eGFR, use of glucose-, BP- and lipid-lowering drugs, and use of tobacco and alcohol.

**Table 3:** Associations of wPRS, based on LD *r*^2^ threshold of 0.2 during selection of SNPs, with incident cardiovascular–kidney complications in the HKDR cohort of 2313 individuals with type 2 diabetes

Model	Continuous swPRS	Top 10% vs bottom 10% wPRS	Top 20% vs bottom 20% wPRS	Top 30% vs bottom 30% wPRS
Association of wPRS with incident CVD				
Model 1^a^				
*N*	2308	459	920	1382
HR (95% CI)	1.15 (1.05, 1.25)	1.72 (1.17, 2.52)	1.72 (1.29, 2.29)	1.21 (0.96, 1.51)
	0.002**	0.006**	<0.001***	0.103
Model 2^b^				
*N*	2218	435	881	1327
HR (95% CI)	1.15 (1.06, 1.26)	1.72 (1.16, 2.55)	1.77 (1.32, 2.38)	1.23 (0.98, 1.55)
*p* value	0.002**	0.007**	<0.001***	0.077
Model 3^c^				
*N*	2218	435	881	1327
HR (95% CI)	1.16 (1.06, 1.27)	1.71 (1.15, 2.54)	1.85 (1.37, 2.49)	1.27 (1.01, 1.60)
*p* value	0.001**	0.008**	<0.001***	0.045*
Model 4^d^				
*N*	2200	430	873	1318
HR (95% CI)	1.16 (1.06, 1.27)	1.67 (1.12, 2.48)	1.87 (1.38, 2.52)	1.26 (1.00, 1.59)
*p* value	0.001**	0.012*	<0.001***	0.050*
Association of wPRS with incident CKD				
Model 1^a^				
*N*	2308	459	920	1382
HR (95% CI)	1.08 (1.01, 1.15)	1.41 (1.04, 1.90)	1.26 (1.02, 1.55)	1.11 (0.94, 1.32)
*p* value	0.023*	0.026*	0.031*	0.223
Model 2^b^				
*N*	2218	435	881	1327
HR (95% CI)	1.09 (1.01, 1.16)	1.41 (1.02, 1.93)	1.31 (1.05, 1.63)	1.17 (0.98, 1.40)
*p* value	0.017*	0.035*	0.015*	0.077
Model 3^c^				
*N*	2218	435	881	1327
HR (95% CI)	1.08 (1.01, 1.16)	1.40 (1.02, 1.93)	1.32 (1.06, 1.64))	1.17 (0.98, 1.39)
*p* value	0.018*	0.038*	0.013*	0.083
Model 4^d^				
*N*	2200	430	873	1318
HR (95% CI)	1.09 (1.02, 1.16)	1.42 (1.03, 1.95)	1.34 (1.07, 1.66)	1.17 (0.98, 1.40)
*p* value	0.013*	0.034*	0.010*	0.074
Association of wPRS with incident cardiovascular–kidney disease				
Model 1^a^				
*N*	2308	459	920	1382
HR (95% CI)	1.09 (1.02, 1.15)	1.48 (1.13, 1.94)	1.30 (1.07, 1.57)	1.11 (0.95, 1.30)
*p* value	0.007**	0.005**	0.008**	0.182
Model 2^b^				
*N*	2218	435	881	1327
HR (95% CI)	1.09 (1.03, 1.16)	1.48 (1.11, 1.96)	1.35 (1.11, 1.65)	1.16 (0.99, 1.37)
*p* value	0.005**	0.007**	0.003**	0.062
Model 3^c^				
*N*	2218	435	881	1327
HR (95% CI)	1.09 (1.03, 1.16)	1.47 (1.10, 1.96)	1.39 (1.14, 1.69)	1.17 (1.00, 1.38)
*p* value	0.004**	0.009**	0.001**	0.051
Model 4^d^				
*N*	2200	430	873	1318
HR (95% CI)	1.10 (1.03, 1.16)	1.45 (1.09, 1.94)	1.41 (1.15, 1.72)	1.19 (1.01, 1.39)
*p* value	0.003**	0.011*	<0.001***	0.038*

^a^Model 1: Adjusted for first principal component (PC1), second principal component (PC2), age, sex, BMI and DD

^b^Model 2: Model 1 + adjusted for metabolic control (HbA_1c_, systolic BP, triacylglycerol, HDL-cholesterol, LDL-cholesterol and eGFR)

^c^Model 3: Model 2 + adjusted for medication use (oral glucose-lowering drugs, insulin, antihypertensives, lipid-regulating drugs)

^d^Model 4: Model 3 + adjusted for tobacco and alcohol use; in this model, the HR of disease duration of diabetes for incident cardiovascular–kidney disease was 1.014 (95% CI 1.002, 1.025, *p*=0.020)

*p<0.05; **p<0.01; ***p<0.001

Analysis of individual components of cardiovascular–kidney events (Table 3) revealed that the per-SD increase in wPRS was associated with 16% higher risk of CVD (HR 1.16 [95% CI 1.06, 1.27], *p*=0.001). The top-20%-wPRS group had an HR of 1.87 (95% CI 1.38, 2.52, *p*<0.001) for a cardiovascular event compared with the bottom-20%-wPRS group. For each component of the cardiovascular events, the swPRS was associated with incident CHD (HR 1.21 [95% CI 1.07, 1.36], *p*=0.003) but not with stroke (HR 1.00 [95% CI 0.86, 1.16], *p*=0.99), PVD (HR 1.06 [95% CI 0.80, 1.39], *p*=0.68) or CHF (HR 1.08 [95% CI 0.89, 1.31], *p*=0.44) (ESM Table 6). For kidney outcomes, the per-SD increase in wPRS was associated with an HR of 1.09 (95% CI 1.02, 1.16, *p*=0.013) for CKD, with the top-20%-wPRS group having an HR of 1.34 (95% CI 1.07, 1.66, *p*=0.010) compared with the bottom-20%-wPRS group (Table 3). The swPRS was not associated with incident ESKD (HR 0.95 [95% CI 0.76, 1.19], *p*=0.66) (ESM Table 6). There was no significant interaction between disease duration and wPRS (both continuous swPRS and top-20%-wPRS vs bottom-20%-wPRS) for CVD, CKD and combined events.

### Association of risk category, stratified by disease duration and wPRS, with incident cardiovascular–kidney events in individuals with type 2 diabetes

We explored the importance of the wPRS relative to diabetes duration (DD) as a risk factor of diabetes-related complications [4]. Per-year increase in baseline DD was independently associated with an HR of 1.014 (95% CI 1.002, 1.025, *p*=0.020) for cardiovascular–kidney complications in the fully adjusted model (Model 4, Table 3). We stratified the HKDR cohort into six groups by wPRS (top 20% and bottom 20%) and baseline DD (<5 years, 5 to <10 years, and ≥10 years). We compared the risk association of these six groups with incident complications and explored whether those with top-20%-wPRS plus short baseline DD of less than 5–10 years would have comparable or higher risk of complications than the bottom-20%-wPRS plus long baseline DD ≥10 years group.

By Kaplan–Meier estimation (ESM Fig. 2, *p*<0.01 in all logrank tests), the ‘top-20%-wPRS plus baseline DD<5 years’ group with a median age of 49.0 (IQR 42.7–59.2) years had a cumulative incidence of 27% for CVD after 20 years, compared with 22% in the ‘bottom-20%-wPRS group plus baseline DD≥10 years’ group with a median age of 59.3 (IQR 51.2–63.8) years. Similarly, 55% and 66% of the ‘top-20%-wPRS plus baseline DD 5 to <10 years’ group with a median age of 54.5 (IQR 47.3–62.4) years developed CKD and cardiovascular–kidney events after 20 years, respectively, compared with 46% and 53% in the ‘bottom 20%-wPRS plus baseline DD≥10 years’ group. However, the ‘top-20%-wPRS plus baseline DD<5 years’ group had a lower cumulative incidence of CKD (38%) and cardiovascular–kidney events (47%) than the ‘bottom 20%-wPRS plus baseline DD≥10 years’ group after 20 years.

Tables 4 and 5 show the unadjusted and adjusted HRs, respectively, for cardiovascular–kidney disease and its components in the six groups. ESM Fig. 3 shows the associated one-minus-survival function curves. Using Cox regression with the ‘bottom-20%-wPRS plus baseline DD<5 years’ group as the referent, the ‘top-20%-wPRS plus baseline DD<5 years’ group had an unadjusted HR of 1.87 (95% CI 1.23, 2.85, *p*=0.003) for CVD vs 1.43 (95% CI 0.81, 2.54, *p*=0.217) in the ‘bottom-20%-wPRS plus baseline DD≥10 years’ group. The ‘top-20%-wPRS plus baseline DD 5 to <10 years’ group had an unadjusted HR of 1.62 (95% CI 1.15, 2.28, *p*=0.006) and 1.60 (95% CI 1.17, 2.19, *p*=0.003) for CKD and cardiovascular–kidney disease, respectively, vs 1.29 (95% CI 0.86, 1.92, *p*=0.216) and 1.38 (95% CI 0.97, 1.98, *p*=0.075) in the ‘bottom-20%-wPRS plus baseline DD≥10 years’ group. The ‘top-20%-wPRS plus baseline DD<5 years’ group had unadjusted HR of 1.04 (95% CI 0.76, 1.42, *p*=0.812) and 1.11 (95% CI 0.84, 1.46, *p*=0.471) for CKD and cardiovascular–kidney events, respectively.

**Table 4:** Association of risk categories based on DD at baseline and wPRS with cardiovascular–kidney disease in prospective HKDR (unadjusted)

Risk category	No. of individuals	HR	95% CI	*p* value
CVD				
Bottom 20% wPRS and disease duration <5 years	230	Reference	Reference	Reference
Bottom 20% wPRS and disease duration 5 to <10 years	130	1.46	0.87, 2.43	0.149
Bottom 20% wPRS and disease duration ≥10 years	102	1.43	0.81, 2.54	0.217
Top 20% wPRS and disease duration <5 years	241	1.87	1.23, 2.85	0.003**
Top 20% wPRS and disease duration 5 to <10 years	116	1.96	1.20, 3.20	0.007**
Top 20% wPRS and disease duration ≥10 years	105	2.47	1.52, 4.02	<0.001***
CKD				
Bottom 20% wPRS and disease duration <5 years	230	Reference	Reference	Reference
Bottom 20% wPRS and disease duration 5 to <10 years	130	1.38	0.97, 1.94	0.071
Bottom 20% wPRS and disease duration ≥10 years	102	1.29	0.86, 1.92	0.216
Top 20% wPRS and disease duration <5 years	241	1.04	0.76, 1.42	0.812
Top 20% wPRS and disease duration 5 to <10 years	116	1.62	1.15, 2.28	0.006**
Top 20% wPRS and disease duration ≥10 years	105	1.99	1.40, 2.83	<0.001***
Cardiovascular–kidney disease				
Bottom 20% wPRS and disease duration <5 years	230	Reference	Reference	Reference
Bottom 20% wPRS and disease duration 5 to <10 years	130	1.21	0.88, 1.68	0.241
Bottom 20% wPRS and disease duration ≥10 years	102	1.38	0.97, 1.98	0.075
Top 20% wPRS and disease duration <5 years	241	1.11	0.84, 1.46	0.471
Top 20% wPRS and disease duration 5 to <10 years	116	1.60	1.17, 2.19	0.003**
Top 20% wPRS and disease duration ≥10 years	105	1.96	1.42, 2.70	<0.001***

**p<0.01; ***p<0.001

**Table 5:** Association of risk categories based on DD at baseline and wPRS with CVD in prospective HKDR (adjusted for covariates)

Risk category	No. of individuals	HR	95% CI	*p* value
CVD				
Bottom 20% wPRS and disease duration <5 years	213	Reference	Reference	Reference
Bottom 20% wPRS and disease duration 5 to <10 years	121	1.31	0.75, 2.23	0.349
Bottom 20% wPRS and disease duration ≥10 years	101	1.28	0.70, 2.34	0.427
Top 20% wPRS and disease duration <5 years	228	2.08	1.34, 3.24	0.001**
Top 20% wPRS and disease duration 5 to <10 years	108	2.05	1.21, 3.47	0.007**
Top 20% wPRS and disease duration ≥10 years	102	2.38	1.40, 4.05	0.001**
CKD				
Bottom 20% wPRS and disease duration <5 years	213	Reference	Reference	Reference
Bottom 20%wPRS and disease duration 5 to <10 years	121	1.08	0.73, 1.59	0.707
Bottom 20% wPRS and disease duration ≥10 years	101	1.00	0.65, 1.54	0.986
Top 20% wPRS and disease duration <5 years	228	1.13	0.81, 1.57	0.478
Top 20% wPRS and disease duration 5 to <10 years	108	1.63	1.12, 2.36	0.010*
Top 20% wPRS and disease duration ≥10 years	102	1.60	1.09, 2.36	0.016*
Cardiovascular–kidney disease				
Bottom 20% wPRS and disease duration <5 years	213	Reference	Reference	Reference
Bottom 20% wPRS and disease duration 5 to <10 years	121	0.92	0.64, 1.32	0.636
Bottom 20% wPRS and disease duration ≥10 years	101	1.06	0.72, 1.57	0.752
Top 20% wPRS and disease duration <5 years	228	1.22	0.90, 1.64	0.196
Top 20% wPRS and disease duration 5 to <10 years	108	1.62	1.16, 2.26	0.005 **
Top 20% wPRS and disease duration ≥10 years	102	1.52	1.07, 2.16	0.020 *

HRs are adjusted for first principal component (PC1), second principal component (PC2), age, sex, BMI, metabolic control (HbA_1c_, systolic BP, triacylglycerol, HDL-cholesterol, LDL-cholesterol and eGFR), medication use (oral glucose-lowering drugs, insulin, antihypertensives, lipid-regulating drugs), and tobacco and alcohol use

*p<0.05; **p<0.01

The results were similar after adjusting for baseline covariates. The ‘top-20%-wPRS plus baseline DD<5 years’ group had an adjusted HR of 2.08 (95% CI 1.34, 3.24, *p*=0.001) for CVD compared with 1.28 (95% CI 0.70, 2.34, *p*=0.427) in the ‘bottom-20%-wPRS plus baseline DD≥10 years’ group. The ‘top-20%-wPRS plus baseline DD 5 to <10 years’ group had an adjusted HR of 1.63 (95% CI 1.12, 2.36, *p*=0.010) and 1.62 (95% CI 1.16, 2.26, *p*=0.005) for CKD and combined cardiovascular–kidney disease, respectively, compared with 1.00 (95% CI 0.65, 1.54, *p*=0.986) and 1.06 (95% CI 0.72, 1.57, *p*=0.752) in the ‘bottom-20%-wPRS plus baseline DD≥10 years’ group. (The ‘top-20%-wPRS plus baseline DD<5 years’ group had an adjusted HR of 1.13 (95% CI 0.81, 1.57, *p*=0.478) and 1.22 (95% CI 0.90, 1.64, *p*=0.196) for CKD and cardiovascular–kidney events, respectively.)

We repeated the analysis by restructuring the ‘bottom-20%-wPRS plus baseline DD≥10 years’ as the reference group where the ‘top-20%-wPRS plus baseline DD 5 to <10 years’ group had an adjusted HR of 1.62 (95% CI 1.04, 2.53, *p*=0.033) and 1.52 (95% CI 1.02, 2.27, *p*=0.040) for CKD and cardiovascular–kidney disease, respectively, while the ‘top-20%-wPRS plus baseline DD<5 years’ group had an adjusted HR of 1.63 (95% CI 0.93, 2.87, *p*=0.089) for CVD, with borderline significance.

### Secondary analysis: association of the wPRS with beta cell function and incident diabetes in people without diabetes in the BHBHK-HKFDS

We examined the associations of the wPRS with beta cell function and 12 year risk of incident diabetes in the BHBHK-HKFDS. swPRS were negatively associated with beta cell function indices and positively associated with risk of incident diabetes with an adjusted OR of 1.37 (95% CI 0.89, 2.12, *p*=0.150), albeit short of significance (ESM Table 7a).

## Discussion

Based on prior knowledge regarding the functions of MDG, we successfully constructed and validated a wPRS, derived from common variants of MDG, which was associated with YOD and incident cardiovascular–kidney complication in type 2 diabetes. After nearly two decades of follow-up, the top-20%-wPRS group with less than 5–10 years of DD at baseline had higher risk of cardiovascular–kidney complication and its components than the bottom-20%-wPRS group with baseline DD ≥10 years. Our findings highlight the potential utility of information of MDG common variants in increasing the precision of risk stratification for early treatment intensification to delay onset of diabetes and its complications.

### Known importance of MDG in diabetes

Genetic and experimental studies demonstrated that rare variants (RV) in MDG could cause abnormal beta cell or adipose biology resulting in familial YOD or syndromic diabetes with high penetrance [1–3]. Aside from monogenic diabetes, RV of MDG increased the risk of the common form of type 2 diabetes. In the largest WES study, including 20,791 individuals with type 2 diabetes and 24,440 individuals without diabetes from five ancestries, RV located in *PDX1* (encoding for pancreatic and duodenal homeobox 1), *GCK* (encoding for glucokinase) and *HNF1A* (encoding for hepatic nuclear factor 1α) were associated with 1.5- to 3.5-increased odds of type 2 diabetes by weighted burden testing [17]. Some of the risk loci for type 2 diabetes were common variants of MDG, despite their small effect size [5, 18, 19]. Type 2 diabetes is a polygenic disease due to common variants and RVs implicated in pancreatic, adipose and muscle biology [19]. In family-based cohort studies, common variants of MDG were shown to modulate the age of diagnosis of MODY. For example, the common *HNF1A* variant I27L advanced age of diagnosis of the protein-truncating subtype of *HNF1A*-MODY [20]. On the other hand, the polygenic risk score for the common form of type 2 diabetes also jointly advanced the age of diagnosis of *HNF1A*-MODY [21]. We and others reported that common variants of type 2 diabetes, including some in MDG, predicted younger age of diagnosis of type 2 diabetes and earlier insulin requirement in both white European and Asian individuals after adjusting for clinical covariables [22, 23].

### Distinct genetic profile of YOD from LOD

Many research groups have reported the heterogeneous phenotypes and aggressive clinical course of YOD including in Chinese individuals [24–26], although large-scale genetic association studies specific to YOD are lacking. Most studies examined the association of known risk variants of type 2 diabetes with age of diagnosis of diabetes or YOD instead of creating a discovery cohort of YOD cases vs either healthy or LOD control individuals. In our study, we used WES data to construct a wPRS for YOD including estimation of effect size of all common variants within MDG from a case–control cohort of YOD vs non-YOD. This was followed by validation and testing of the association of the wPRS with incident cardiovascular–kidney complications in independent Chinese cohorts. We and others had reported that different ages of diagnosis might be attributed to differences in genetics. In the Botnia Family Study, heritability (h^2^) of type 2 diabetes was 0.69 in people diagnosed at age 35–60 years and dropped to 0.31 when including those diagnosed at up to age 75 years [27]. In Pima Indians with high prevalence of YOD, there was bimodal distribution of 2 h plasma glucose with strong heritability of acute insulin secretion and body fat [28]. In Hong Kong Chinese individuals, family history of YOD was associated with six- to eightfold increased risk of diabetes vs <1.6-fold for family history of diabetes diagnosed after the age of 50 years compared with no family history of diabetes [11].

In a small-scale GWAS study, we first reported the genetic association of *DACH1*, a transcription factor, with familial YOD in Chinese with replication in a multi-ethnic Asian population [29]. This SNP, implicated in insulin secretion and islet development, was also associated with systolic BP, insulin resistance and CVD in the Chinese population. Using familial YOD as a discovery cohort, we first discovered its genetic association with *PAX4*, another transcription factor, with replication in other Asian cohorts [30]. Likewise, using a pathway approach, SNPs located in *CPE* (encoding for carboxypeptidase E) and *IDE* (encoding for insulin-degrading enzyme), implicated in human islet amyloid biology, were associated with YOD with replication in Asians [31]. These findings supported the utility of our prospective cohorts with extensive phenotypes and the importance of genetic factors in YOD in Chinese individuals. The latter had lower beta cell function and more rapid decline with disease duration than LOD [26].

In a GWAS of 24,986 cases of type 2 diabetes and 187,130 controls in the UK Biobank stratified by age of diagnosis, there were subgroup-specific type 2 diabetes risk loci and subgroup-specific effect size of the common variants [7]. Seventeen independent SNPs had different effect size in different age of diagnosis subgroups where SNPs mapped to *SLCO4C1*, *SLC6A1*, *RP11–58B2.1*, *PAM* and *CCND2-AS1* were more strongly associated with cases diagnosed before the age of 50 years than with cases diagnosed after the age of 70 years, although none of them were traditional MDG. The gene encoding for peptidylglycine α-amidating monooxygenase (*PAM*) was recently identified as a novel MDG [32]. In another UK Biobank analysis of type 2 diabetes stratified by BMI and age of diagnosis (BMI>30 kg/m^2^, BMI<30 kg/m^2^ and age of diagnosis <60 years, BMI<30 kg/m^2^ and age of diagnosis >60 years), 277 lead SNPs were identified with 18 of them, including one in *NEUROG3* (encoding for neurogenin-3), showing subgroup difference [8]. *NEUROG3* mutations can cause permanent neonatal diabetes and childhood-onset diabetes with severe insulin deficiency [33]. Similarly, Swedish researchers used cluster analysis to categorise individuals with diabetes by various combinations of age, age of diagnosis, BMI, HOMA-B, HOMA-IR and autoantibodies. These clusters had different patterns of genetic factors with prognostic significance for insulin requirement and CKD [34–36]. Taken together, the phenotypic heterogeneity and high risk of complications in individuals with YOD, with likely distinct genetic background from LOD, call for more precise classification and stratification for early and personalised prevention [4, 37].

### Risk stratification by MDG wPRS

Our study using MDG wPRS has filled some of this knowledge gap. Our discovery cohort included only individuals with YOD as cases, in contrast to most discovery cohorts that include type 2 diabetes cases irrespective of age of diagnosis. Based on a candidate gene approach, we used all variants and their corresponding effect estimates to construct a wPRS for YOD with independent validation. In the main analysis, the wPRS was associated with YOD and incident cardiovascular–kidney events. The top-20%-wPRS group had 87%, 34% and 41% higher risk of incident CVD, CKD and composite outcomes, respectively, compared with the lowest-20%-wPRS group.

Although DD is a key driving factor of diabetes complications due to accumulating glycaemic burden and other factors, the MDG wPRS was no less, if not more, important than DD for risk stratification. Our data showed that those with the top 20% risk by genetics, despite short baseline DD (<5 to 10 years), had a higher hazard of cardiovascular–kidney complications than those in the bottom 20% with long baseline DD≥10 years. Since MDG are implicated in beta cell biology, insulin resistance and other syndromic features, we postulated that poor glycaemic trajectory and hence rapid accumulation of glycaemic burden within a short period might advance these complications.

More detailed analysis showed that the high genetic risk group with short DD were of younger age and had more ‘optimal’ glycaemic control with HbA_1c_ <53.0 mmol/mol (7%) than the low genetic risk group with long DD at baseline (ESM Table 8). This might give an illusion of a low-risk profile to their attending physicians in routine care settings where biogenetic markers are not yet in clinical use and might lead to less-frequent monitoring of glycaemic control and incident complications. This low level of vigilance could mean missed opportunity for early intensification of glucose-lowering therapy, control of other risk factors and introduction of organ-protective therapy. Of note, individuals in the high genetic risk group with short DD had higher BMI, BP, dyslipidaemia and were more likely to be active smokers than the low genetic risk group with long DD. More studies are needed to confirm the use of genetic markers to identify high-risk individuals and use this personalised information to inform practice and motivate behavioural change. To this end, in a 1 year RCT of 420 Chinese individuals with type 2 diabetes, provision of information on their genetic risks for complications improved empowerment and reduced distress, albeit without effect on metabolic control [38].

### Limitations and prospects

In this study, we utilised prospective cohorts enriched with YOD and clinical events to ascertain the utility of the MDG wPRS. To our knowledge, there are no comparable prospective cohorts in the Chinese population for external replication, which is one of the weaknesses, and the results might not be generalisable to other ethnicities. We do not have sufficient sample size to obtain precise estimates of effect size and confirm the significance of individual SNPs. The limited sample size also disabled hypothesis-free discovery of variants across the genome to derive an inclusive wPRS for YOD, and large-scale studies are required. Similarly, we did not perform sex-specific analysis in the discovery cohort for derivation of sex-specific wPRS in view of the limited sample size. As the wPRS was not sex-specific, it was applied to analysis of the whole validation cohort for YOD and HKDR prospective cohort for cardiovascular–kidney complications. Nevertheless, sex was included as a covariate for adjustment across all main analyses. Whether sex-specific wPRS would have different performance from non-sex-specific wPRS in this regard requires further exploration. Existing genetic discovery studies for type 2 diabetes involve mostly individuals with LOD. Based on the potential difference in genetics of YOD and LOD, we speculated that the general type 2 diabetes polygenic risk scores derived from these cohorts might be less efficient in predicting YOD compared with a YOD polygenic risk score. Comparative performance in other traits, including glycaemic deterioration and complications, would require further examination.

### Conclusion

Common variants of MDG were associated with YOD and cardiovascular–kidney complications in type 2 diabetes in Chinese individuals. Availability of the genetic information might help improve risk stratification for primary or secondary prevention purposes.

## Supplementary Information

Below is the link to the electronic supplementary material.

ESM (PDF 1789 KB)

## Data Availability

Due to local law and regulation, no data can be shared with external parties. Summary statistics may be shared upon reasonable request to the corresponding author.

## Abbreviations

BHBHK: Better Health for Better Hong Kong
CHF: Congestive heart failure
CKD: Chronic kidney disease
CUHK: Chinese University of Hong Kong
DD: Diabetes duration
ESKD: End-stage kidney disease
GWAS: Genome-wide association studies
HKDR: Hong Kong Diabetes Register
HKFDS: Hong Kong Family Diabetes Study
LD: Linkage disequilibrium
LOD: Later-onset diabetes
MDG: Monogenic diabetes genes
PVD: Peripheral vascular disease
PWH: Prince of Wales Hospital
RV: Rare variants
swPRS: Standardised wPRS
WES: Whole-exome sequencing
wPRS: Weighted polygenic risk score
YOD: Young-onset diabetes

## Author’s relationships and activities

JCNC has received research grants (through institutions) and/or honoraria for consultancy and/or giving lectures from Applied Therapeutics, AstraZeneca, Bayer, Boehringer Ingelheim, Eli Lilly, Hua Medicine, Powder Pharmaceuticals, Merck Serono, MSD, Pfizer, Sanofi, Viatris and Zuellig Pharma. AOYL has served as an advisory committee member for AstraZeneca, Boehringer Ingelheim, Sanofi and Amgen, and has received research grants and travel grants from AstraZeneca, Boehringer Ingelheim, MSD, Novartis, Novo Nordisk, Sanofi and Amgen. RCWM has received research grants for clinical trials from AstraZeneca, Bayer, MSD, Novo Nordisk, Sanofi, Roche and Tricida and honoraria for consultancy or lectures from AstraZeneca and Boehringer Ingelheim. APSK has received research grants and/or speaker honoraria from Abbott, AstraZeneca, Bayer, Boehringer Ingelheim, Eli Lilly, Merck Serono, Nestle, Novo Nordisk and Sanofi. JCNC, WYS and RCWM hold patents for using biogenetic markers to predict risk of diabetes and its complications. JCNC, CKPL, RCWM and WYS are co-founders of GemVCare, a biotech start-up supported by the Technology Start-up Support Scheme for Universities of the Hong Kong Government Innovation and Technology Commission. RCWM is an advisory board member of the editorial board of Diabetologia. The authors declare that there are no other relationships or activities that might bias, or be perceived to bias, their work.
