# Supplementary material for: A polygenic risk score derived from common variants of monogenic diabetes genes is associated with young-onset type 2 diabetes and cardiovascular–kidney complications
Source: Diabetologia. 2024 Nov 23;68(2):367–81. doi: 10.1007/s00125-024-06320-3 (PMC11732898; doi:10.1007/s00125-024-06320-3)
Supplement: Supplementary file 1 — ESM (PDF 1789 KB) [file 125_2024_6320_MOESM1_ESM.pdf]

Contents

ESM Table 1. List of the 34 monogenic diabetes genes selected in this study....................................2

ESM Table 2a. Number of single-nucleotide polymorphisms used for construction of weighted polygenic risk scores based on three different linkage disequilibrium thresholds................................4

ESM Table 2b. Single-nucleotide polymorphisms for construction of the weighted polygenic risk score based on linkage disequilibrium r^2 threshold of 0.2 6

ESM Table 3. Details of whole-exome sequencing and genotyping data. 13

ESM Table 4. Definitions of cardiovascular-kidney events associated with diabetes.........................14

ESM Table 5a. Performance of three weighted polygenic risk scores (wPRS) based on different linkage disequilibrium r^2 threshold during selection of single-nucleotide polymorphisms in validation cohort 15

ESM Table 5b. Performance of the weighted polygenic risk scores (wPRS) based on linkage disequilibrium r^2 threshold of 0.2 in sensitivity analyses of the validation cohort.............................16

ESM Table 6. Associations of wPRS, based on LD r2 threshold of 0.2 during selection of SNPs, with components of incident cardiovascular–kidney complications in the HKDR cohort of 2313 individuals with type 2 diabetes...17

ESM Table 7a. Association of standardized weighted polygenic risk score (swPRS) with indices of beta-cell function, insulin resistance and incident diabetes at 12 years in the community-based BHBHK-HKFDS cohort of participants free of diabetes at baseline 18

ESM Table 7b. Association of standardized weighted polygenic risk score (swPRS) with indices of beta-cell function, insulin resistance and incident diabetes at 12 years in the community-based BHBHK-HKFDS cohort of participants free of diabetes at baseline (women only, with availability of information of history of pregnancy and diabetes in pregnancy) 19

ESM Table 8a. Baseline characteristics of [Lowest 20% wPRS and disease duration (DD) at baseline ≥10 years] group versus [Top 20% wPRS and DD at baseline <5 years] group 20

ESM Table 8b. Baseline characteristics of [Lowest 20% wPRS and disease duration (DD) at baseline ≥10 years] group versus [Top 20% wPRS and DD at baseline <10 years] group 22

ESM Figure 1. Visualization of distribution of the subjects from different cohorts in the study.........24

ESM Figure 2. Kaplan-Meier estimation with associated one-minus-survival functions for incident cardiovascular-kidney events stratified by disease duration and weighted polygenic risk score (wPRS) rank...31

ESM Figure 3. Cumulative incidence and hazards of incident cardiovascular-kidney complications in patients stratified by wPRS and disease duration...32

ESM Table 1. List of the 34 monogenic diabetes genes selected in this study

Gene	Subtypes	Gene	Subtypes	Gene	Subtypes
HNF1A	MODY	***DCAF17***	Syndrome	***PDX1***	NDM, MODY
HNF1B	NDM, MODY, Syndrome	***DNAJC3***	Syndrome	***PIK3R1***	Syndrome
HNF4A	MODY	***DYRK1B***	Syndrome	***PLIN1***	IR
GCK	NDM, MODY	***GATA4***	NDM, Syndrome	***POLD1***	IR
PAX4	MODY	***GATA6***	NDM, Syndrome	***PPARG***	IR
WFS1	NDM, Syndrome	***INSR***	NDM, IR	***PPP1R15B***	Syndrome
SLC29A3	NDM, Syndrome	***KCNJ11***	NDM, MODY	***RFX6***	NDM, MODY
ABCC8	NDM, MODY	***LMNA***	IR	***TRMT10A***	Syndrome
AKT2	IR	***NEUROD1***	NDM, MODY	***ZBTB20***	Syndrome
APPL1	MODY	***PAX6***	Syndrome	***ZFP57***	NDM
CEL	MODY, Syndrome	***PCBD1***	Syndrome	***INS***	NDM, MODY
CISD2	NDM, Syndrome				

Abbreviations: maturity-onset diabetes of the young, MODY; insulin resistance, IR; neonatal diabetes mellitus, NDM

Hepatocyte nuclear factor-1 alpha, HNF1A
Hepatocyte nuclear factor-1 beta, HNF1B
Hepatocyte nuclear factor 4 alpha, HNF4A

Glucokinase, GCK
Paired Box 4, PAX4
Wolframin ER Transmembrane Glycoprotein, WFS1
Solute Carrier Family 29 Member 3, SLC29A3
ATP Binding Cassette Subfamily C Member 8, ABCC8
AKT Serine/Threonine Kinase 2, AKT2
Adaptor Protein, Phosphotyrosine Interacting With PH Domain And Leucine Zipper 1, APPL1
Carboxyl Ester Lipase, CEL
CDGSH Iron Sulfur Domain 2, CISD2
DDB1 And CUL4 Associated Factor 17, DCAF17
DnaJ Heat Shock Protein Family (Hsp40) Member C3, DNAJC3
Dual Specificity Tyrosine Phosphorylation Regulated Kinase 1B, DYRK1B
GATA Binding Protein 4, GATA4
GATA Binding Protein 6, GATA6
Insulin Receptor, INSR
*Potassium Inwardly Rectifying Channel Subfamily J Member 11, KCNJ11**
Lamin A/C, LMNA
*Neurogenic differentiation 1, NEUROD1**
Paired Box 6, PAX6
Pterin-4 Alpha-Carbinolamine Dehydratase 1, PCBD1
*pancreatic and duodenal homeobox 1, PDX1**
Phosphoinositide-3-Kinase Regulatory Subunit 1, PIK3R1
Perilipin 1, PLIN1
*DNA polymerase delta 1, POLD1**
Peroxisome proliferator activated receptor gamma, PPARG
Protein phosphatase 1 regulatory subunit 15B, PPP1R15B
Regulatory Factor X6, RFX6
TRNA Methyltransferase 10A, TRMT10A
Zinc Finger And BTB Domain Containing 20, ZBTB20
Zinc finger protein 57 homolog, ZFP57
Insulin, INS

In view of the increasing discovery of monogenic diabetes genes with different levels of evidence, we did not include all and the most updated list of monogenic diabetes genes. The list of monogenic diabetes genes here is consistent with our recently published epidemiological studies for evaluating the prevalence of monogenic diabetes and associated risk of complications and mortality, and randomized controlled trial for evaluating the usefulness and cost-effectiveness of including genetic information in clinical practice (1,2).

(1) Tsoi STF, Lim C, Ma RCW, et al. Monogenic diabetes in a Chinese population with young-onset diabetes: A 17-year prospective follow-up study in Hong Kong. *Diabetes Metab Res Rev*. 2024;40(5):e3823. doi:10.1002/dmrr.3823
(2) O CK, Fan YN, Fan B, et al. Precision Medicine to Redefine Insulin Secretion and Monogenic Diabetes-Randomized Controlled Trial (PRISM-RCT) in Chinese patients with young-onset diabetes: design, methods and baseline characteristics. *BMJ Open Diabetes Res Care*. 2024;12(3):e004120. Published 2024 Jun 19. doi:10.1136/bmjdrc-2024-004120

ESM Table 2a. Number of single-nucleotide polymorphisms used for construction of weighted polygenic risk scores based on three different linkage disequilibrium thresholds

**Linkage disequilibrium threshold, r^2 0.2**		**Linkage disequilibrium threshold, r^2 0.4**		**Linkage disequilibrium threshold, r^2 0.6**	
**Gene**	**No. of SNPs**	**Gene**	**No. of SNPs**	**Gene**	**No. of SNPs**
HNF1A	5	*HNF1A*	5	*HNF1A*	6
HNF1B	4	*HNF1B*	8	*HNF1B*	8
HNF4A	10	*HNF4A*	12	*HNF4A*	15
GCK	6	*GCK*	7	*GCK*	7
PAX4	3	*PAX4*	5	*PAX4*	6
WFS1	3	*WFS1*	5	*WFS1*	6
SLC29A3	5	*SLC29A3*	5	*SLC29A3*	7
ABCC8	15	*ABCC8*	21	*ABCC8*	27
AKT2	3	*AKT2*	4	*AKT2*	5
APPL1	2	*APPL1*	2	*APPL1*	2
CEL	3	*CEL*	3	*CEL*	4
CISD2	2	*CISD2*	2	*CISD2*	2
DCAF17	5	*DCAF17*	5	*DCAF17*	5
DNAJC3	2	*DNAJC3*	3	*DNAJC3*	3
DYRK1B	2	*DYRK1B*	3	*DYRK1B*	4
GATA4	4	*GATA4*	7	*GATA4*	10
GATA6	4	*GATA6*	6	*GATA6*	6
INSR	21	*INSR*	27	*INSR*	32

KCNJ11	1	*KCNJ11*	1	*KCNJ11*	1
LMNA	1	*LMNA*	1	*LMNA*	1
NEUROD1	1	*NEUROD1*	1	*NEUROD1*	1
PAX6	3	*PAX6*	4	*PAX6*	4
PCBD1	1	*PCBD1*	1	*PCBD1*	1
PDX1	2	*PDX1*	2	*PDX1*	2
PIK3R1	5	*PIK3R1*	8	*PIK3R1*	11
PLIN1	3	*PLIN1*	4	*PLIN1*	5
POLD1	3	*POLD1*	4	*POLD1*	5
PPARG	4	*PPARG*	6	*PPARG*	6
PPP1R15B	1	*PPP1R15B*	1	*PPP1R15B*	1
RFX6	3	*RFX6*	3	*RFX6*	4
TRMT10A	3	*TRMT10A*	4	*TRMT10A*	4
ZBTB20	1	*ZBTB20*	1	*ZBTB20*	1
ZFP57	3	*ZFP57*	3	*ZFP57*	3
INS	1	*INS*	1	*INS*	1
**Total**	**135**	**Total**	**175**	**Total**	**206**

Abbreviations: single-nucleotide polymorphisms, SNP

ESM Table 2b. Single-nucleotide polymorphisms for construction of the weighted polygenic risk score based on linkage disequilibrium r^2 threshold of 0.2

**Gene**	**SNP_ID**	**Risk Allele**	**Odds Ratio**
HNF1A	rs1169288	C	1.25
	rs55783344	T	1.12
	rs76048388	T	1.00
	rs1169307	C	1.47
	rs11065390	G	1.00
HNF1B	rs3110641	G	1.04
	rs2269843	C	1.10
	rs17847523	T	1.30
	rs4239217	G	1.32
HNF4A	rs2071197	G	1.10
	rs113182233	T	1.10
	rs2071199	C	1.55
	rs2868095	G	1.01
	rs745975	C	1.18
	rs1800961	T	1.69
	rs11574739	A	1.04
	rs193127978	C	1.42

	rs3818247	G	1.02
	rs41280258	G	1.22
GCK	rs13306388	C	1.07
	rs186758907	A	1.05
	rs117651210	C	1.83
	rs2268575	T	1.05
	rs2268573	G	1.08
	rs2971677	A	1.13
PAX4	rs712700	T	1.20
	rs2233580	T	1.54
	rs3824004	G	1.02
WFS1	rs181377234	T	1.93
	rs4629534	T	1.35
	rs1805070	A	1.42
SLC29A3	rs2277257	G	1.09
	rs77626641	C	1.30
	rs883764	A	1.04
	rs1084004	T	1.22
	rs780680	T	1.01
ABCC8	rs41282912	C	1.15

	rs739689	A	1.10
	rs4757513	G	1.04
	rs2077655	A	1.08
	rs1805036	G	1.30
	rs4148626	A	1.30
	rs2074315	A	1.14
	rs73423065	G	1.18
	rs1799857	G	1.03
	rs8192694	G	1.14
	rs76948244	G	1.05
	rs886290	T	1.09
	rs2074317	C	1.08
	rs2301704	C	1.71
	rs77889556	G	1.01
AKT2	rs28362958	C	1.06
	rs3730051	C	1.29
	rs73933241	G	1.20
APPL1	rs76173303	A	1.03
	rs1533272	T	1.16
CEL	rs592267	T	1.28

	rs488087	C	1.06
	rs117460641	G	1.09
CISD2	rs2198199	G	1.45
	rs223333	C	1.02
DCAF17	rs76137360	T	2.03
	rs142462938	C	1.40
	rs201495882	C	1.09
	rs3731980	C	1.54
	rs2356781	G	1.15
DNAJC3	rs142956355	G	1.13
	rs2289813	G	1.03
DYRK1B	rs56110449	A	1.10
	rs11083539	T	1.58
GATA4	rs573733348	A	1.34
	rs809205	C	1.21
	rs1062219	T	1.19
	rs884662	C	1.24
GATA6	rs3764504	C	1.03
	rs115998024	C	1.31
	rs3764962	A	1.28

	rs1941084	G	1.27
INSR	rs1051690	T	1.29
	rs78312382	A	1.05
	rs13306448	T	1.31
	rs13306447	G	1.16
	rs11085212	C	1.21
	rs4031066	T	1.02
	rs117592694	A	1.42
	rs9789298	C	1.26
	rs16994213	T	1.10
	rs41509747	A	1.13
	rs2352955	A	1.21
	rs10419596	A	1.30
	rs2303672	T	1.51
	rs45596032	C	1.34
	rs11085213	G	1.03
	rs117123940	C	1.36
	rs2229428	C	2.33
	rs891087	A	1.18
	rs6510976	T	1.03

	rs3745544	G	1.10
	rs140204110	G	2.61
KCNJ11	rs5219	T	1.25
LMNA	rs547915	T	1.63
NEUROD1	rs1801262	T	1.01
PAX6	rs3026384	G	1.02
	rs3026371	C	1.02
	rs4440995	G	1.12
PCBD1	rs9712	C	1.04
PDX1	rs9581943	G	1.13
	rs73169687	G	1.29
PIK3R1	rs706714	A	1.00
	rs2302976	G	1.17
	rs171649	G	1.11
	rs3730082	G	1.17
	rs3730089	A	1.27
PLIN1	rs76899769	T	1.09
	rs1077903	A	1.38
	rs12906281	T	1.10
POLD1	rs1673026	G	1.24

	rs3218768	T	1.40
	rs3218776	C	1.01
PPARG	rs2120825	G	1.31
	rs13306745	G	1.13
	rs4684103	C	1.06
	rs3856806	T	1.03
PPP1R15B	rs145021449	C	1.33
RFX6	rs11754683	C	1.16
	rs2184343	T	1.05
	rs643943	C	1.06
TRMT10A	rs1054730	T	1.12
	rs80053092	T	1.03
	rs73832830	T	1.20
ZBTB20	rs17670486	C	1.24
ZFP57	rs28454267	A	2.32
	rs2840215	G	1.17
	rs375984	C	1.12
INS	rs3842756	T	1.45

ESM Table 3. Details of whole-exome sequencing and genotyping data.

Data processing and quality control of the Whole-exome sequencing (WES) data	Genotyping and imputation data of the validation and testing cohorts	Harmonization of data from different arrays
All subjects in the discovery cohort underwent WES by Illumina HiSeq X™ Ten Sequencing System with paired-end run of 151 cycles per read, which were conducted at the Broad Institute in Cambridge, MA, USA. Details of the library preparation and sequencing had been published with variants calling and quality control (QC) performed in-house. Quality control of sequencing reads was conducted by FastQC (version 0.11.9, https://www.bioinformatics.babraham.ac.uk/projects/fastqc/). We aligned sequencing reads to the human reference genome (GRCh38 with decoy and HLA contigs) in an alt-aware manner using Burrows-Wheeler Aligner (bwa) -mem2 software (v2.2.1). PCR duplicates were marked using the Picard tools (http://broadinstitute.github.io/picard/). The Genome Analysis Toolkit (GATK) best practice protocol for germline short variant discovery was adopted for quality recalibration and genotype calling for SNPs (Single Nucleotide Polymorphisms) and Indels (small Insertion and Deletions) when base quality recalibration was conducted using the "known sites" files from the GATK resource bundle (https://console.cloud.google.com/storage/browser/genomics-public-data/resources/broad/hg38/v0).<br>Joint calling, which is based on the genetic information of the whole cohort, was used for variants calling. To control the quality of called variants, we firstly applied GATK (v4.1.9.0) Variant Quality Score Recalibration to calculate discrimination algorithms based on the variants' characteristics for filtering variants classified as poor quality. Genotype refinement was then performed on the called variants. Finally, we filtered variants by genotype quality and depth. Cleaned variants was annotated with variant number based on dbSNP155 for downstream analyses.	The validation cohort and testing cohorts came from the HKFDS, BHBHK and HKDR cohorts not involved in the WES study. Subjects recruited from the HKDR underwent genotyping using Illumina® Omni2.5 + Exome Array, whereas those recruited from HKFDS were genotyped by Infinium® Global Screening Array. Subjects without diabetes were genotyped using either Illumina® Omni2.5+Exome Array or Asian Screening Array. The per-individual QC of genotype data consists of four steps: (1) sex-checking based on the genotype call from chromosome X; (2) detection of low-quality samples based on call rate and heterozygosity rate; (3) detection of possible familial relationship or duplicated individuals using estimates of identity-by-descent (IBD); (4) detection of population stratification by performing principal component (PC) analysis. Only biallelic autosomal SNPs were included in the per-marker QC. SNPs were excluded from further analysis if (1) Hardy–Weinberg equilibrium (HWE) $p<1\times10^{-4}$ and (2) minor allele frequency (MAF)<1%; or 3) call rate<95%. In particular, SNPs with MAF≥1% but ≤5% were excluded if their call rate was <99%. Within each individual cohort, we imputed the genotype data to the 1000 Genomes Project phase III reference panel (October 2014) using the Michigan Imputation Server.	After standard procedure of data processing and quality control for the whole exome sequencing (WES) data from discovery cohort, we firstly transferred the genome build of WES data from assembly GRCh38 to GRCh37 by liftOver tool (https://genome.ucsc.edu/cgi-bin/hgLiftOver). We excluded insertion and deletion variations. Only biallelic SNPs were included for downstream analysis. Then, we extracted common variants within ± 1000 base pairs of the gene region of the 34 MODY genes (MDG). We used 1000 genome project of East Asian populations as a reference to estimate the linkage disequilibrium statistics r2 and clumped variants for each gene region based on three thresholds of r^2: 0.2, 0.4, 0.6.<br><br>As aforementioned, individuals in the testing and validation cohorts coming from the HKDR, HKFDS and BHBHK were genotyped by three genotyping platforms: Illumina® Omni2.5 + Exome Array, Infinium® Global Screening Array, and Asian Screening Array. Genotype data with good quality were imputed to the same reference panel – 1000 Genomes Project phase III reference panel (October 2014). Only biallelic SNPs in imputed data with imputation quality $r^2 > 0.5$ and minor allele frequency > 0.01 were used for downstream analysis.<br><br>We overlapped the variants from the clumped data of WES and imputation data of HKDR, HKFDS and LKS cohort, and used these overlapped variants to compute the polygenic risk scores in HKDR, HKFDS and BHBHK for testing and validation purpose.<br><br>Given that we performed clumping for WES data first and then overlapped with array data from testing and validation cohorts, we summarized the number of variants within ± 1000 base pairs of the gene region of the 34 MDG in each dataset before clumping. Among common variants that passed the quality control for each dataset, there are 724 variants in WES data, 5068 in imputation data of Omni2.5 + Exome Array data (individuals with diabetes from HKDR and part of individuals without diabetes from LKs cohort), 4656 in imputation data of Global Screening Array (individuals from HKFDS), and 4957 in imputation data of Asian Screening Array (individuals from LKS cohort). A total of 607 variants are available in both WES and genotyping arrays from the three platforms before clumping.<br><br>After clumping WES data by LD statistic r^2 of 0.2 estimating based on 1000 Genomes Project phase III reference panel (October 2014), 199 out of 724 variants remained to make a total of 135 variants in analysis after overlapping with genotyping array data.

ESM Table 4. Definitions of cardiovascular-kidney events associated with diabetes

**Diabetic cardiovascular-kidney events**	**Definition**
Coronary heart disease	ICD-9 Diagnosis Code: 410-414
Stroke	ICD-9 Diagnosis Code: 430-434, 436-438
Peripheral vascular disease	ICD-9 Diagnosis Code: 250.7, 785.4, 443.81, 443.9,<br>ICD-9 Procedure Code: 38.08, 38.18, 38.38, 38.48, 38.68, 38.88, 39.25, 39.49, 39.56, 39.57, 39.58, 39.59, 39.99; 00.55, 17.56, 39.50, 39.79, 84.1, but discharges with a traumatic amputation diagnosis code (895-897) were excluded
Congestive heart failure	ICD-9 Diagnosis Code: 428
Cardiovascular disease	All codes defining coronary artery disease, stroke, peripheral vascular disease, and congestive heart failure
End-stage kidney disease	1) two eGFR <15 mL/min/1.73m^2 separated by at least 90 days<br>2) Hemodialysis dialysis (ICD-9 Procedure Code 39.95) with diagnosis of chronic kidney disease (ICD-9 Diagnosis Code 585) or renal failure (ICD-9 Diagnosis Code 586)<br>3) Peritoneal dialysis (ICD-9 Procedure Code 54.98)<br>4) Transplant of kidney (ICD-9 Procedure Code 55.6)<br>5) Complications of transplanted kidney (ICD-9 Diagnosis Code 996.81)<br>6) Persons with a condition influencing their health status; organ or tissue replaced by transplant; kidney (ICD-9 Diagnosis Code V42.0)
Chronic kidney disease	1) End-stage kidney disease<br>2) two eGFR<60 mL/min/1.73m^2 separated by at least 90 days
Cardiovascular-kidney composite	Comprises all of the above complications

Abbreviations: the International Classification of Diseases, Ninth Revision, ICD-9; estimated glomerular filtration rate, eGFR

ESM Table 5a. Performance of three weighted polygenic risk scores (wPRS) based on different linkage disequilibrium r^2 threshold during selection of single-nucleotide polymorphisms in validation cohort

**Model**	**Standardized weighted polygenic risk score (independent variable) based on LD r^2 threshold of**		
	**0.2**	0.4	0.6
Adjusted for PC1 and PC2	*n* = 5830<br>OR = 1.07 (0.99 to 1.14)<br>*p*-value = 0.077	*n* = 5830<br>OR = 1.06 (0.99 to 1.14)<br>*p*-value = 0.094	*n* = 5830<br>OR = 1.05 (0.98 to 1.13)<br>*p*-value = 0.153
Add-on adjustment for sex	*n* = 5830<br>OR = 1.07 (0.99 to 1.14)<br>*p*-value = 0.082	*n* = 5830<br>OR = 1.06 (0.99 to 1.14)<br>*p*-value = 0.105	*n* = 5830<br>OR = 1.05 (0.98 to 1.13)<br>*p*-value = 0.166
Add-on adjustment for body mass index	*n* = 5799<br>OR = 1.07 (0.99 to 1.15)<br>*p*-value = 0.074	*n* = 5799<br>OR = 1.06 (0.99 to 1.14)<br>*p*-value = 0.088	*n* = 5799<br>OR = 1.05 (0.98 to 1.13)<br>*p*-value = 0.143

Outcome variable: young-onset diabetes
Abbreviations: linkage disequilibrium, LD; number, *n*; odd ratio, OR

ESM Table 5b. Performance of the weighted polygenic risk scores (wPRS) based on linkage disequilibrium r^2 threshold of 0.2 in sensitivity analyses of the validation cohort

**Model**	**Subjection inclusion**		
	**Only non-DM and YOD (Outcome: YOD)**	**Only non-DM and LOD (Outcome: LOD)**	**Only YOD and LOD (Outcome: YOD)**
Unadjusted	*n* = 1160<br>OR = 1.08 (0.94 to 1.25)<br>*p*-value = 0.292	*n* = 4910<br>OR = 1.01 (0.89 to 1.14)<br>*p*-value = 0.914	*n* = 5590<br>OR = 1.07 (1.00 to 1.15)<br>*p*-value = 0.052
Adjusted for PC1 and PC2	*n* = 1160<br>OR = 1.08 (0.93 to 1.24)<br>*p*-value = 0.316	*n* = 4910<br>OR = 1.01 (0.89 to 1.15)<br>*p*-value = 0.876	*n* = 5590<br>OR = 1.07 (0.99 to 1.14)<br>*p*-value = 0.081
Add-on adjustment for sex	*n* = 1160<br>OR = 1.07 (0.93 to 1.24)<br>*p*-value = 0.333	*n* = 4910<br>OR = 1.01 (0.89 to 1.15)<br>*p*-value = 0.873	*n* = 5590<br>OR = 1.07 (0.99 to 1.14)<br>*p*-value = 0.085
Add-on adjustment for body mass index	*n* = 1159<br>OR = 1.08 (0.94 to 1.25)<br>*p*-value = 0.280	*n* = 4880<br>OR = 1.01 (0.89 to 1.14)<br>*p*-value = 0.914	*n* = 5559<br>OR = 1.07 (0.99 to 1.15)<br>*p*-value = 0.078

Abbreviations: number, N; odd ratio, OR

ESM Table 6. Associations of wPRS, based on LD r2 threshold of 0.2 during selection of SNPs, with components of incident cardiovascular–kidney complications in the HKDR cohort of 2313 individuals with type 2 diabetes

**Complications**	***N***	***Hazard ratios***	***p-value***
**Coronary heart disease**	**2200**	**1.21 [95% CI 1.07, 1.36]**	**0.003****
Stroke	2200	1.00 [95% CI 0.86, 1.16]	0.99
Peripheral vascular disease	2200	1.06 [95% CI 0.80, 1.39]	0.68
Congestive heart failure	2200	1.08 [95% CI 0.89, 1.31]	0.44
End-stage kidney disease	2200	0.95 [95% CI 0.76, 1.19]	0.66

Adjusted for PC1, PC2, age, sex, BMI, disease duration of diabetes, metabolic control (HbA1c, systolic BP, triglycerides, HDL-cholesterol, LDL-cholesterol and eGFR), medication use (oral glucose-lowering drugs, insulin, antihypertensives, lipid-regulating drugs), and tobacco and alcohol use

* $p<0.05$; ** $p<0.01$; *** $p<0.001$

ESM Table 7a. Association of standardized weighted polygenic risk score (swPRS) with indices of beta-cell function, insulin resistance and incident diabetes at 12 years in the community-based BHBHK-HKFDS cohort of participants free of diabetes at baseline

**Outcomes**	**Models**	**Number**	**Odd ratios (OR) or Regression coefficients (RC) with 95% CI**
Incident diabetes	Adjusted for PC1, PC2, age and sex	304	OR = 1.11 (0.79 to 1.57), *p*=0.537
	Add-on adjustment of metabolic control#	300	OR = 1.37 (0.89 to 2.12), *p*=0.150
HOMA2-%B	Adjusted for PC1, PC2, age and sex	285	RC = -2.17 (-5.48 to 1.15), *p*=0.200
	Add-on adjustment of metabolic control#	281	RC = -1.02 (-3.56 to 1.53), *p*=0.432
HOMA2-IR	Adjusted for PC1, PC2, age and sex	285	RC = -0.021 (-0.065 to 0.023), *p*=0.356
	Add-on adjustment of metabolic control#	281	RC = -0.015 (-0.052 to 0.021), *p*=0.410
Insulinogenic index	Adjusted for PC1, PC2, age and sex	345	RC = -1.77 (-11.21 to 7.67), *p*=0.712
	Add-on adjustment of metabolic control#	338	RC = -3.06 (-12.1 to 5.97), *p*=0.505
Disposition index	Adjusted for PC1, PC2, age and sex	344	RC = -0.50 (-3.42 to 2.42), *p*=0.736
	Add-on adjustment of metabolic control#	338	RC = -0.71 (-3.65 to 2.23), *p*=0.634

Body mass index, fasting blood glucose, systolic blood pressure, triglycerides, low-density lipoprotein cholesterol, high-density lipoprotein cholesterol. Abbreviations: odd ratio, OR; regression coefficient, RC; homeostasis model assessment of beta-cell function, HOMA2-%B; homeostasis model assessment of insulin resistance, HOMA2-IR

ESM Table 7b. Association of standardized weighted polygenic risk score (swPRS) with indices of beta-cell function, insulin resistance and incident diabetes at 12 years in the community-based BHBHK-HKFDS cohort of participants free of diabetes at baseline (women only, with availability of information of history of pregnancy and diabetes in pregnancy)

Outcomes	Models	Number	Odd ratios (OR) or Regression coefficients (RC) with 95% CI
Incident diabetes	Adjusted for PC1, PC2, age, metabolic control#, number of pregnancy and history of diabetes in pregnancy	168	OR = 1.389 (0.777 to 2.482), *p* = 0.267
HOMA2-%B	Adjusted for PC1, PC2, age, metabolic control#, number of pregnancy and history of diabetes in pregnancy	156	RC = -2.966 (-6.306 to 0.373), *p* = 0.081
HOMA2-IR	Adjusted for PC1, PC2, age, metabolic control#, number of pregnancy and history of diabetes in pregnancy	156	RC = -0.038 (-0.087 to 0.011), *p* = 0.127
Insulinogenic index	Adjusted for PC1, PC2, age, metabolic control#, number of pregnancy and history of diabetes in pregnancy	184	RC = -6.765 (-22.884 to 9.353), *p* = 0.409
Disposition index	Adjusted for PC1, PC2, age, metabolic control#, number of pregnancy and history of diabetes in pregnancy	184	RC = -0.126 (-4.463 to 4.210), *p* = 0.954

Body mass index, fasting blood glucose, systolic blood pressure, triglycerides, low-density lipoprotein cholesterol, high-density lipoprotein cholesterol.
Abbreviations: odd ratio, OR; regression coefficient, RC; homeostasis model assessment of beta-cell function, HOMA2-%B; homeostasis model assessment of insulin resistance, HOMA2-IR

ESM Table 8a. Baseline characteristics of [Lowest 20% wPRS and disease duration (DD) at baseline ≥10 years] group versus [Top 20% wPRS and DD at baseline <5 years] group

**Baseline characteristics**	**Lowest 20% wPRS and DD at baseline ≥10 years**	**Top 20% wPRS and DD at baseline <5 years**	***p*-value**
**Age (years)**	**59.3 (51.2 – 63.8)**	**49.0 (42.7 – 59.2)**	**<0.001*** **
Men (%)	41 (40.2%)	121 (50.2%)	0.090
**Body mass index (kg/m^2)**	**23.6 (21.9 – 25.6)**	**24.8 (22.9 – 27.4)**	**0.002** **
**HbA1c (%)**	**7.4 (6.7 – 8.6)**	**6.8 (6.1 – 8.1)**	**<0.001*** **
**HbA1c (mmol/mol)**	**57 (50-70)**	**51 (43-65)**	
**Fasting plasma glucose (mmol/L)**	**8.4 (6.9 – 10.0)**	**7.3 (6.2 – 9.1)**	**0.005** **
Systolic blood pressure (mmHg)	129 (117 - 140)	128 (118 - 138)	0.507
**Diastolic blood pressure (mmHg)**	**73 ± 10**	**76 ± 10**	**0.011* **
**Triglyceride (mmol/L)**	**1.1 (0.7 – 1.5)**	**1.4 (1.0 – 2.1)**	**<0.001*** **
**HDL cholesterol (mmol/L)**	**1.5 (1.2 – 1.7)**	**1.2 (1.0 – 1.5)**	**<0.001*** **
LDL cholesterol (mmol/L)	3.0 (0.8)	3.1 (0.9)	0.326
Estimated glomerular filtration rate (mL/min/1.73m^2)	92 (80 - 99)	93 (83 - 105)	0.057
**Oral glucose-lowering drugs**	**73 (71.6%)**	**145 (60.2%)**	**0.045* **
**Insulin**	**23 (22.5%)**	**5 (2.1%)**	**<0.001*** **
Anti-hypertensive drugs	30 (29.4%)	61 (25.3%)	0.432
Lipid-regulating drugs	15 (14.7%)	28 (11.6%)	0.430

**Smoking**			**0.045***
**Current smoker**	**11 (10.8%)**	**50 (20.7%)**	
**Ex-smoker**	**16 (15.7%)**	**24 (10.0%)**	
**Non-smoker**	**75 (73.5%)**	**167 (69.3%)**	
Alcohol			0.973
Current drinker	12 (11.8%)	27 (11.3%)	
Ex-drinker	13 (12.7%)	29 (12.1%)	
Non-drinker	77 (75.5%)	184 (76.7%)	

Number (%), mean (SD) or median (IQR)

Abbreviations: glycated haemoglobin, HbA1c; *p<0.05, **p<0.01, ***p<0.001

ESM Table 8b. Baseline characteristics of [Lowest 20% wPRS and disease duration (DD) at baseline ≥10 years] group versus [Top 20% wPRS and DD at baseline <10 years] group

Baseline characteristics	Lowest 20% wPRS and DD at baseline ≥10 years	Top 20% wPRS and DD at baseline <10 years	*p*-value
**Age (years)**	**57.7 (9.8)**	**52.0 (11.9)**	**<0.001**
**Men**	**41 (40.2%)**	**183 (51.3%)**	**0.049**
**Body mass index (kg/m^2)**	**23.6 (21.9 – 25.6)**	**24.7 (22.8 – 27.2)**	**0.003**
**HbA1c (%)**	**7.4 (6.7 – 8.6)**	**6.9 (6.2 – 8.1)**	**<0.001**
**HbA1c (mmol/mol)**	**57 (50-70)**	**52 (44-65)**	
**Fasting plasma glucose (mmol/L)**	**8.4 (6.9 – 10.0)**	**7.5 (6.3 – 9.3)**	**0.017**
Systolic blood pressure (mmHg)	129 (117 - 140)	129 (117 - 139)	0.706
Diastolic blood pressure (mmHg)	74 (66 - 81)	76 (69 - 83)	0.051
**Triglyceride (mmol/L)**	**1.1 (0.7 – 1.5)**	**1.3 (0.9 – 2.1)**	**<0.001**
**HDL cholesterol (mmol/L)**	**1.5 (1.2 – 1.7)**	**1.3 (1.1 – 1.5)**	**<0.001**
LDL cholesterol (mmol/L)	3.0 ± 0.8	3.1 ± 0.9	0.347
Estimated glomerular filtration rate (mL/min/1.73m^2)	92 (80 - 99)	92 (81 - 104)	0.099
Oral glucose-lowering drugs	73 (71.6%)	227 (63.6%)	0.135
**Insulin**	**23 (22.5%)**	**16 (4.5%)**	**<0.001**
Anti-hypertensive drugs	30 (29.4%)	98 (27.5%)	0.697
Lipid-regulating drugs	15 (14.7%)	42 (11.8%)	0.427

Smoking			0.140
Current smoker	11 (10.8%)	66 (18.5%)	
Ex-smoker	16 (15.7%)	42 (11.8%)	
Non-smoker	75 (73.5%)	249 (69.7%)	
Alcohol			0.942
Current drinker	12 (11.8%)	41 (11.5%)	
Ex-drinker	13 (12.7%)	41 (11.5%)	
Non-drinker	77 (75.5%)	273 (76.9%)	

Number (%), mean (SD) or median (IQR)

Abbreviations: glycated haemoglobin, HbA1c; *p<0.05, **p<0.01, ***p<0.001

ESM Figure 1. Visualization of distribution of the subjects from different cohorts in the study

ESM Figure 1a. Participants from the HKDR Cohort

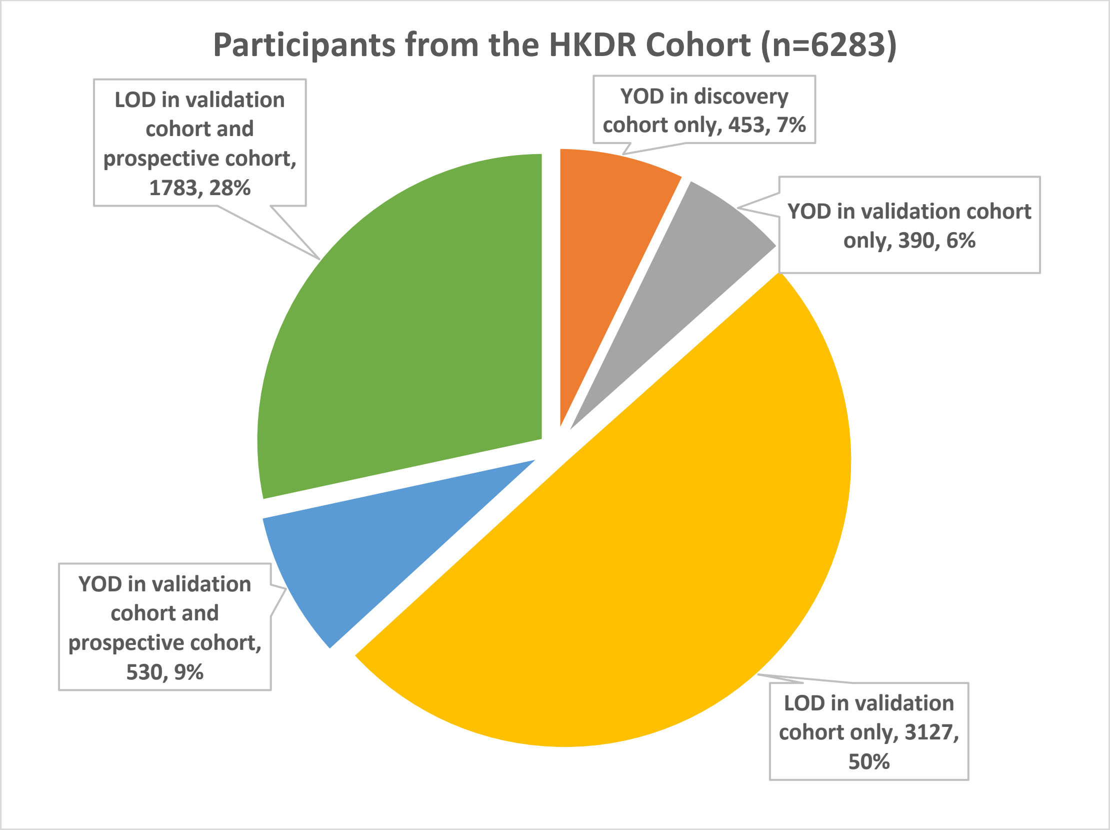

ESM Figure 1b. Participants from the BHBHK Cohort

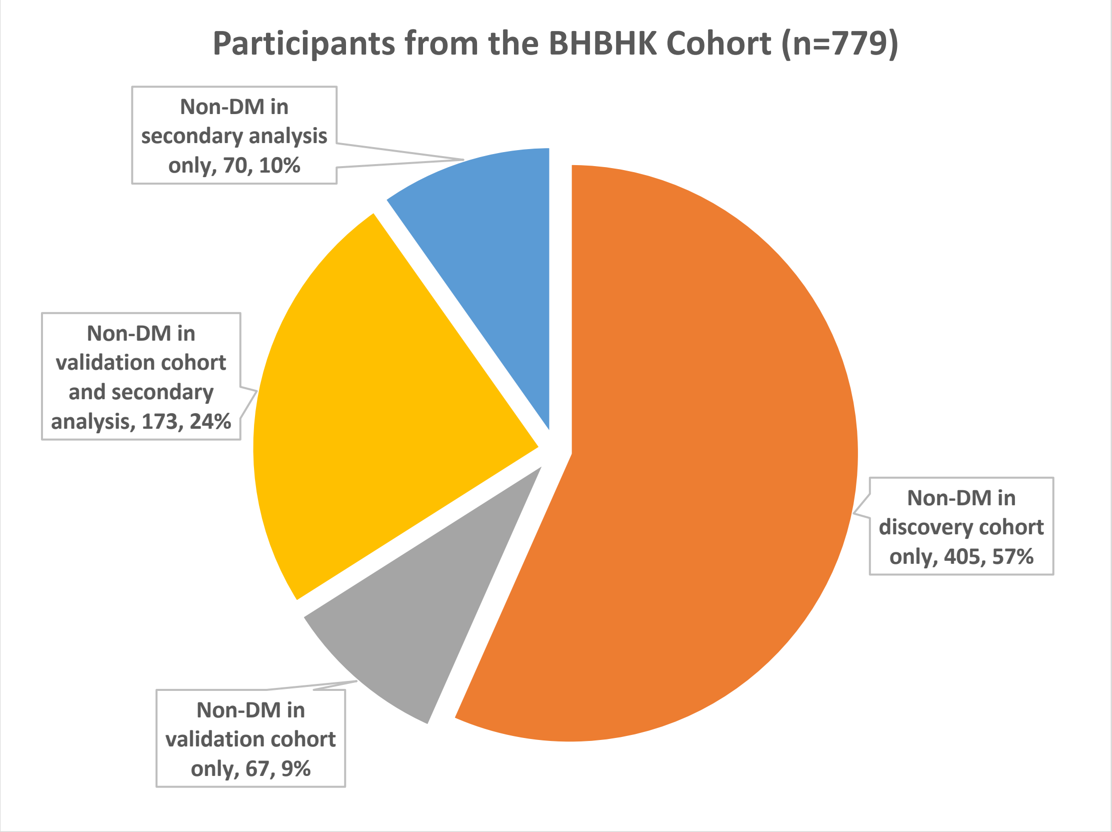

ESM Figure 1c. Participants from the HKFDS Cohort

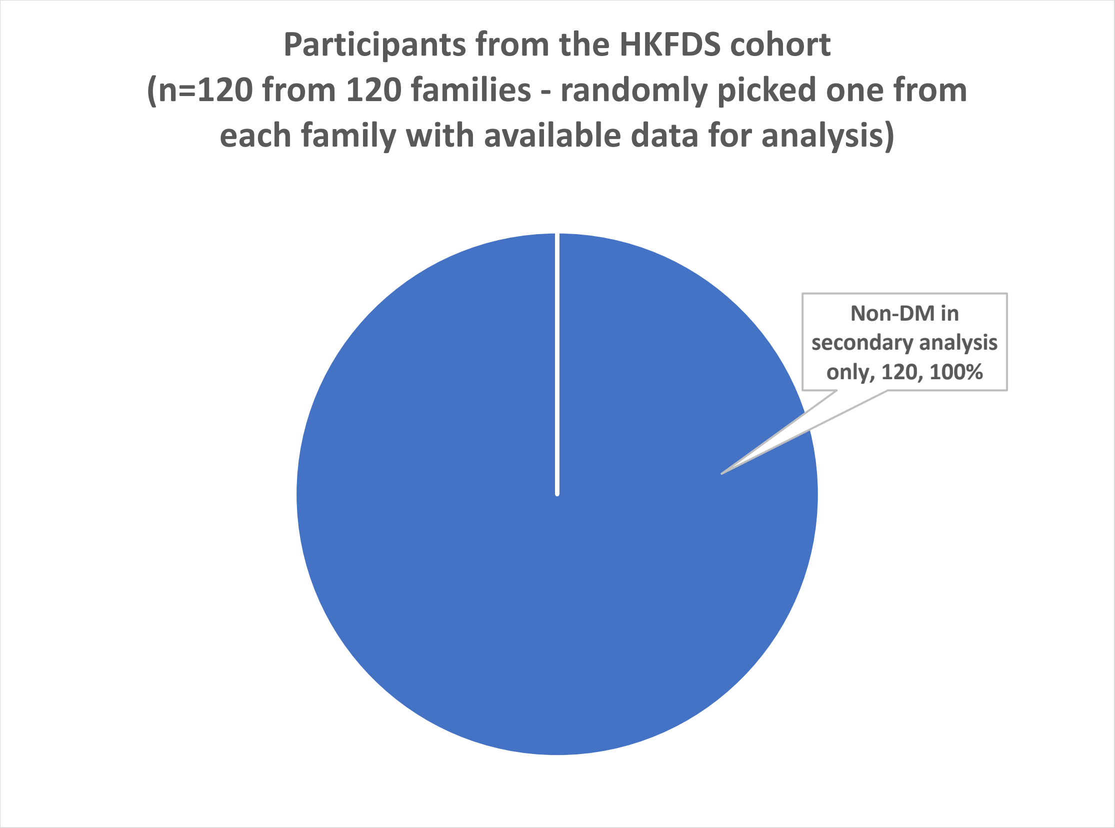

ESM Figure 1d. Participants in the discovery cohort

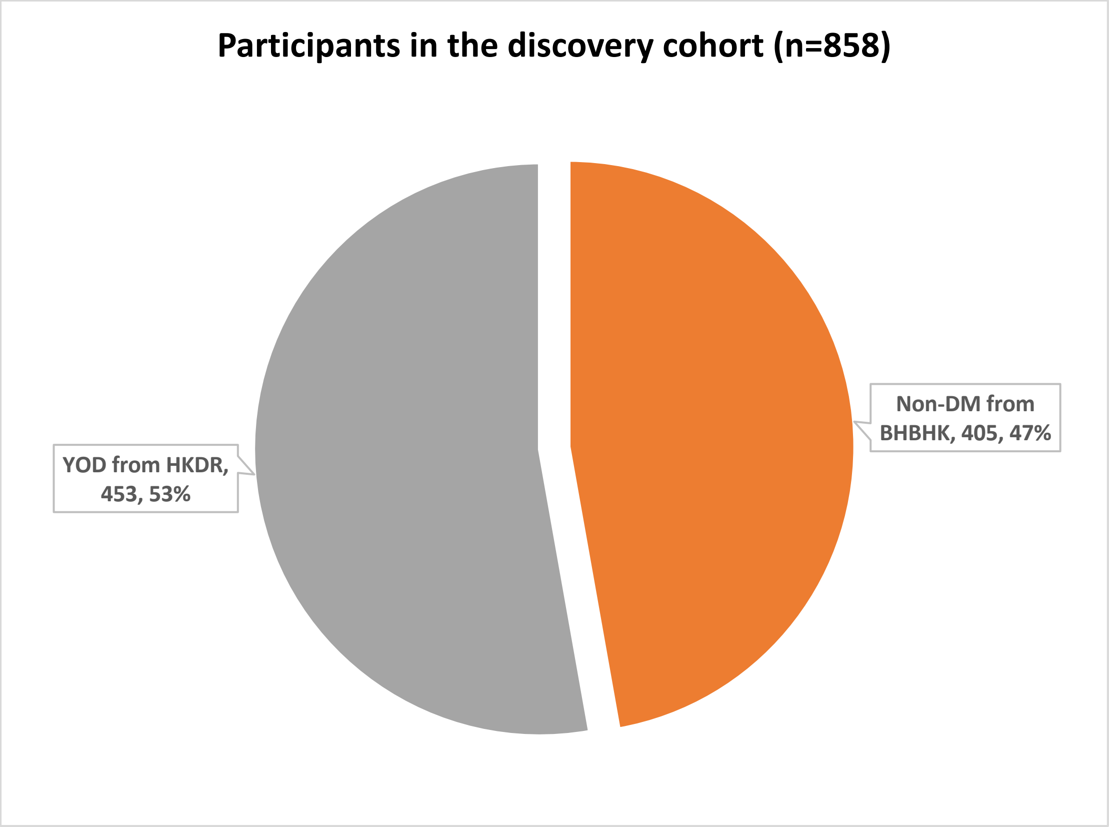

ESM Figure 1e. Participants in the validation cohort

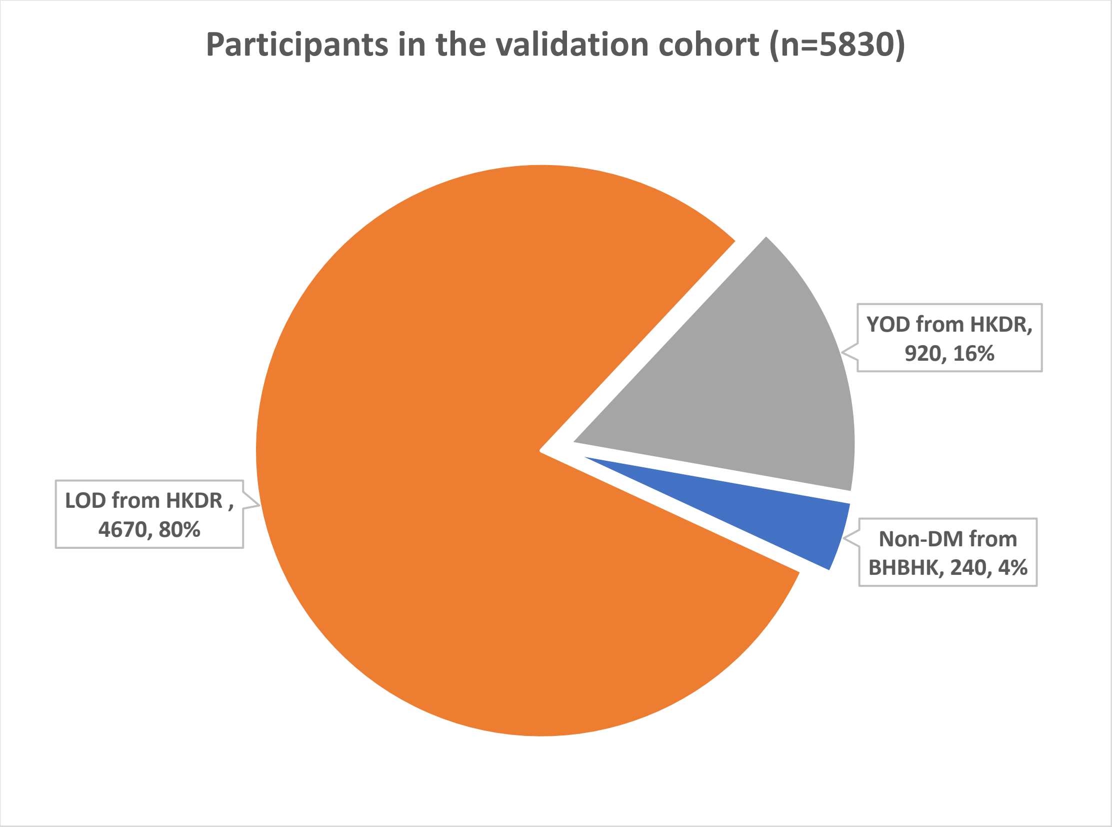

ESM Figure 1f. Participants in the HKDR prospective cohort

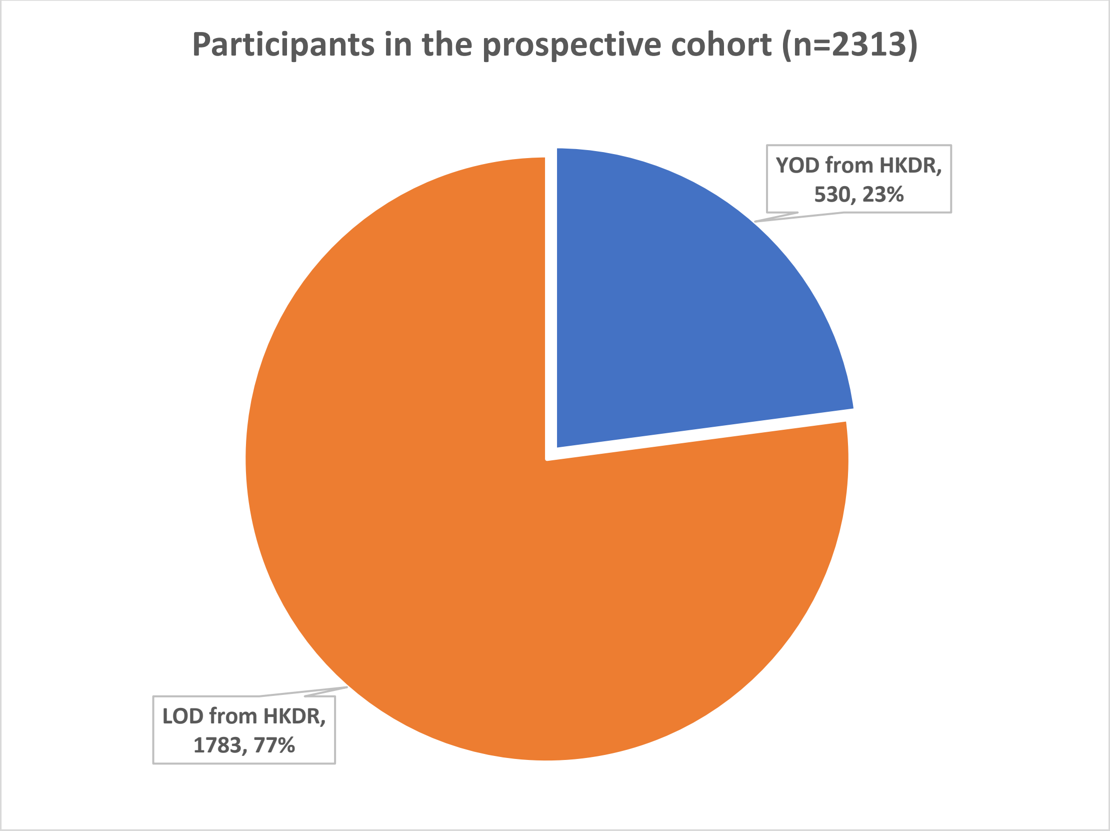

ESM Figure 1g. Participants in the secondary analysis

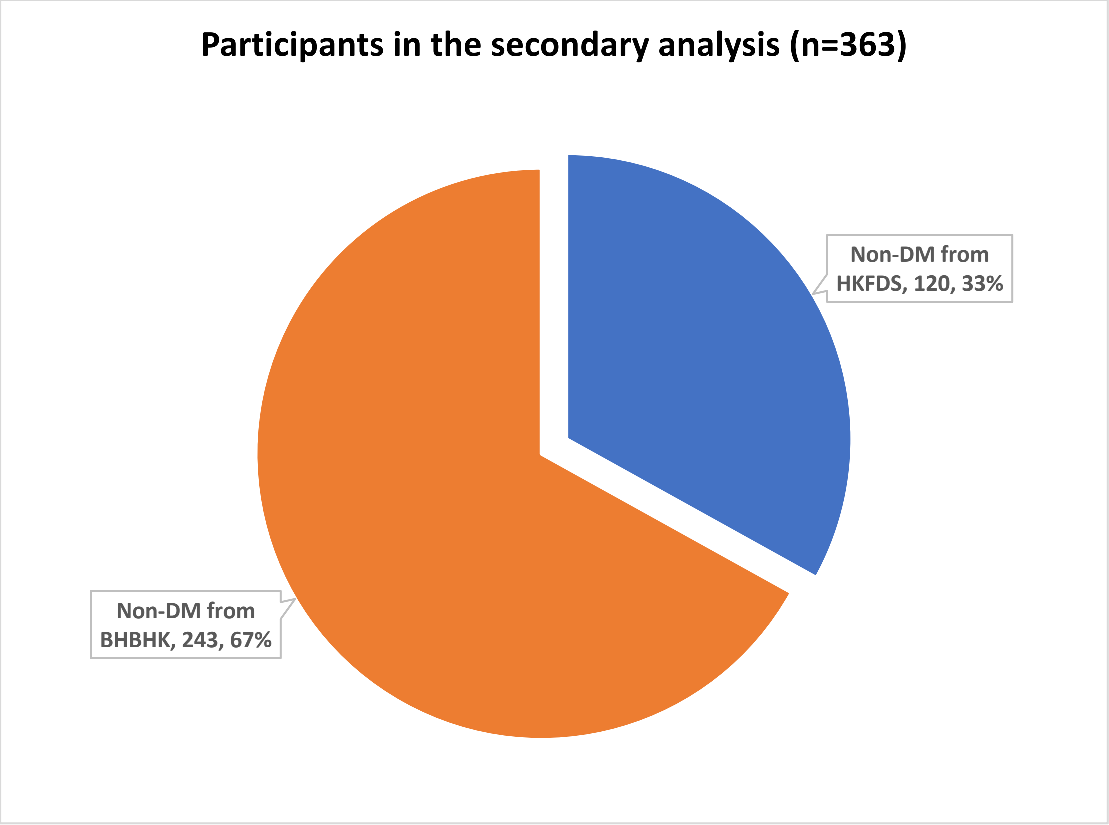

ESM Figure 2. Kaplan-Meier estimation with associated one-minus-survival functions for incident cardiovascular-kidney events stratified by disease duration and weighted polygenic risk score (wPRS) rank

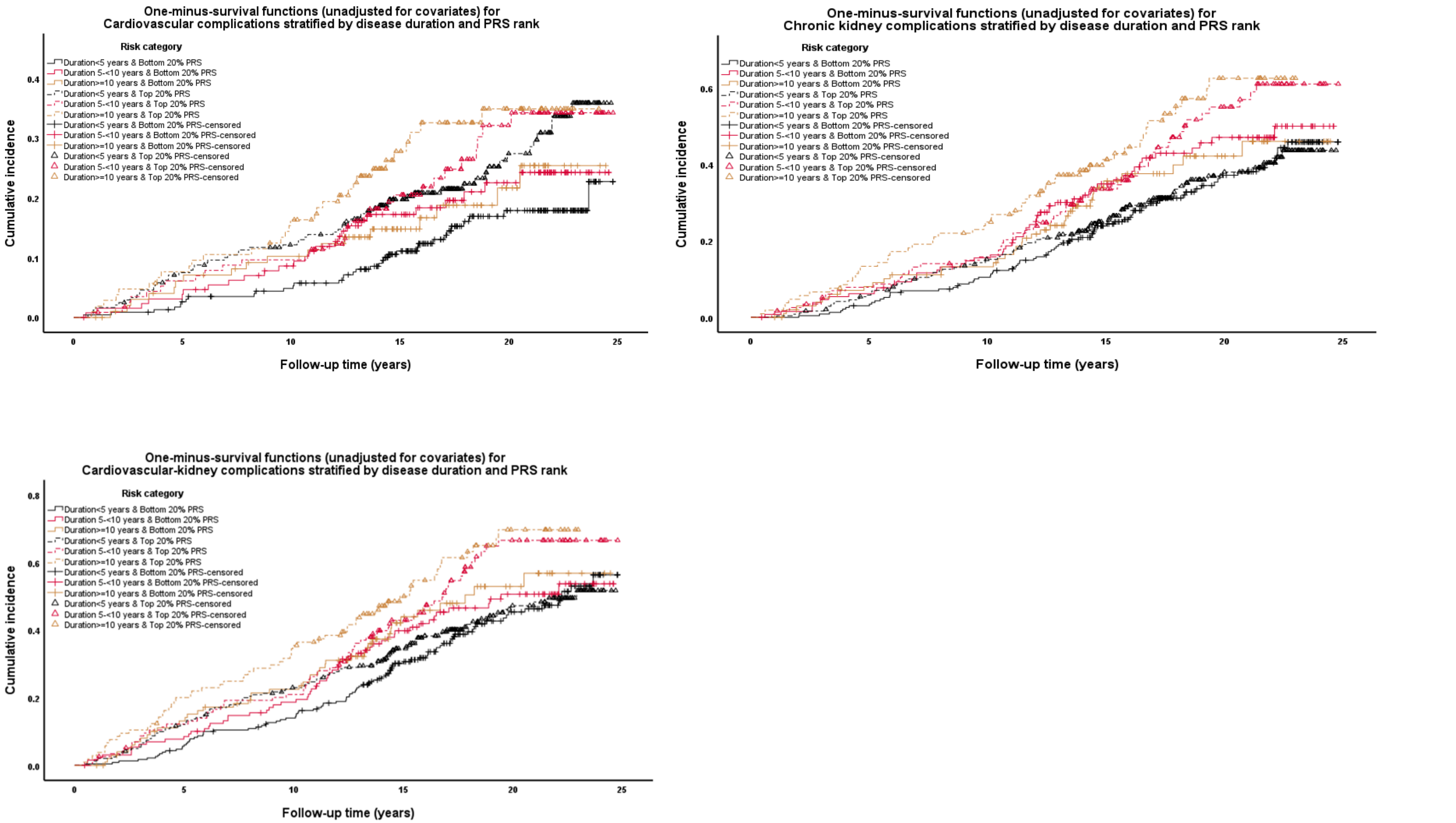

The y-axes refer to the cumulative incidence of the complications ranging from 0 to 1 (or 0% to 100%).

ESM Figure 3. Cumulative incidence and hazards of incident cardiovascular-kidney complications in patients stratified by wPRS and disease duration

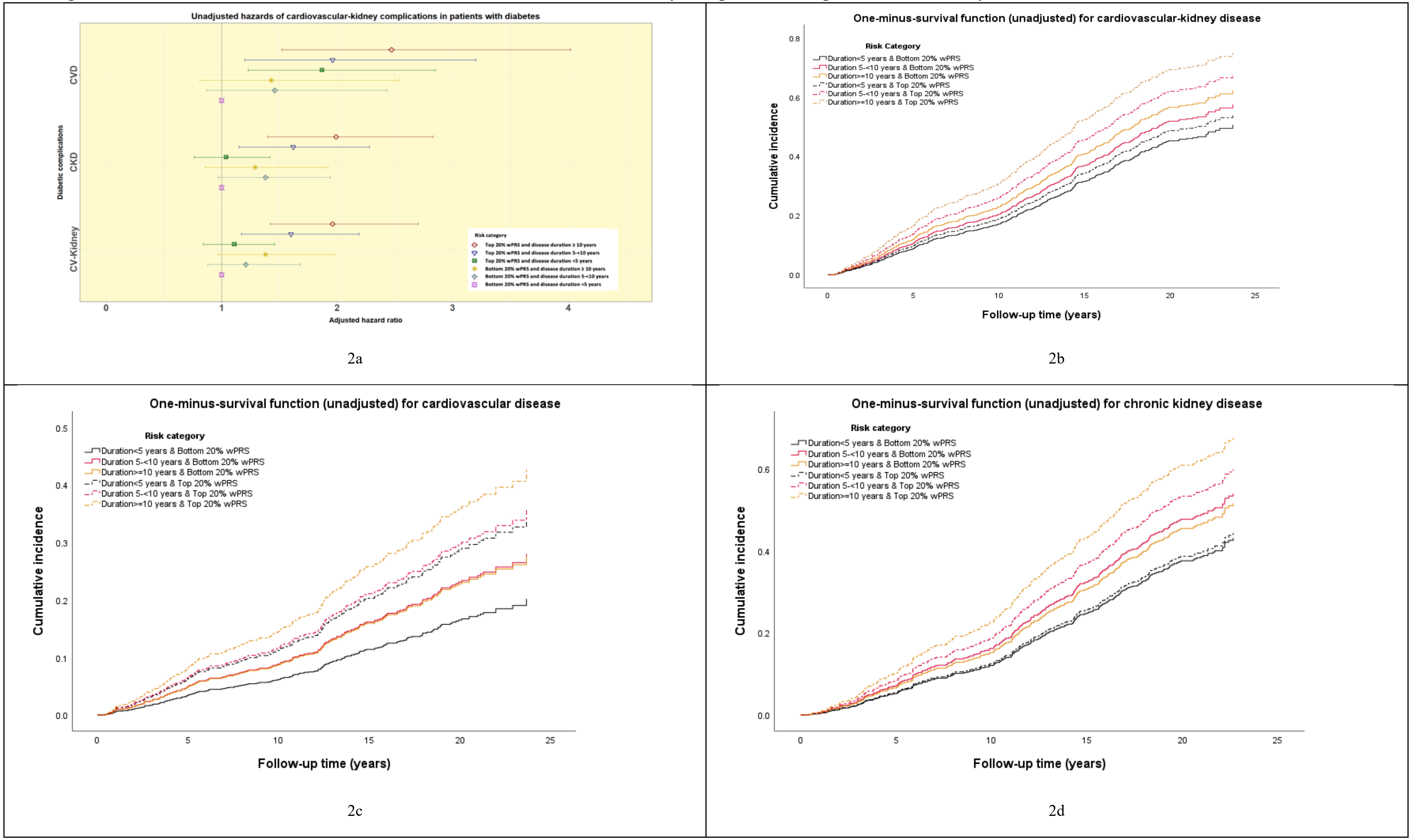

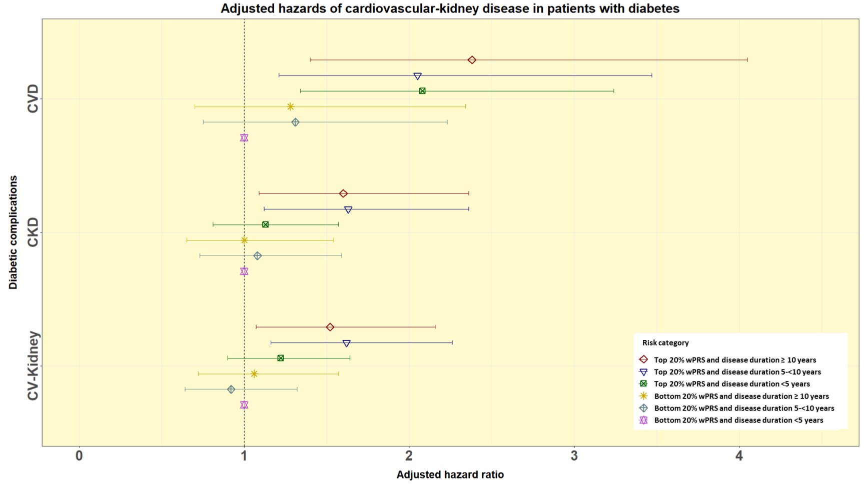

2e

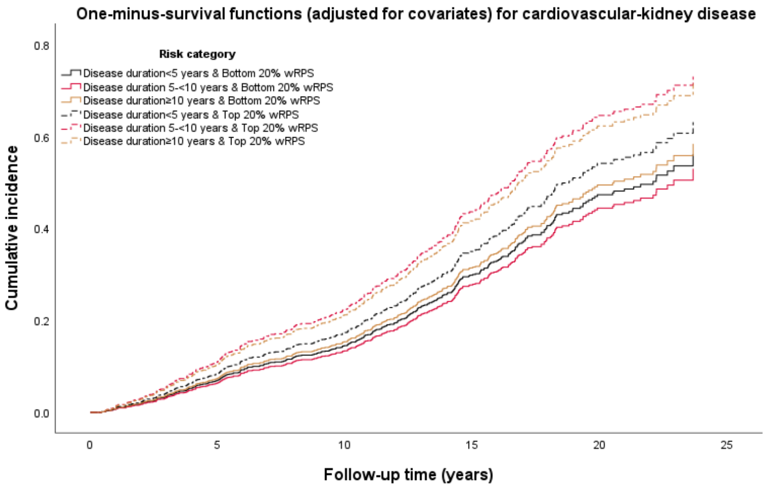

2f

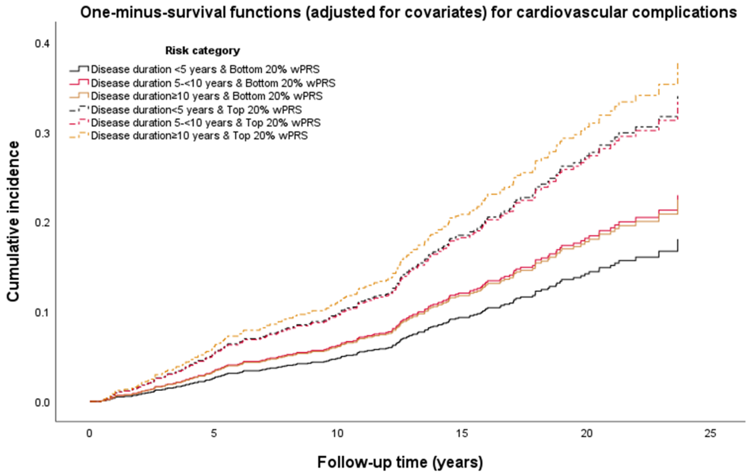

2g

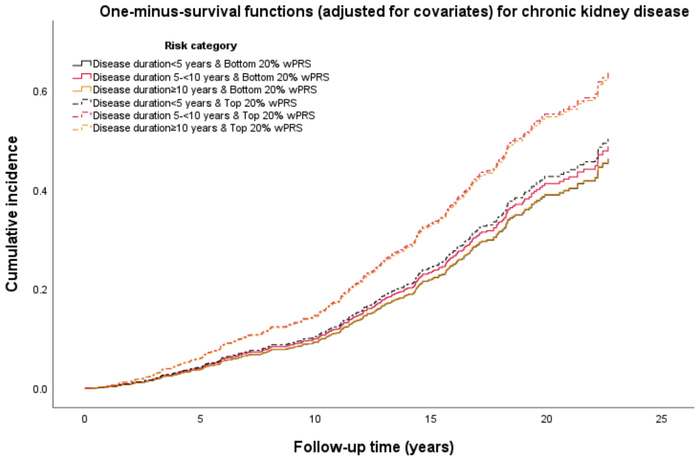

2h

Abbreviations: weighted polygenic risk score, wPRS; cardiovascular disease, CVD; chronic kidney disease, CKD; cardiovascular-kidney, CV-Kidney.
